# Poly(ADP-ribosyl)ation Acts in the DNA Demethylation of Mouse Primordial Germ Cells Also with DNA Damage-Independent Roles

**DOI:** 10.1371/journal.pone.0046927

**Published:** 2012-10-05

**Authors:** Fabio Ciccarone, Francesca Gioia Klinger, Angela Catizone, Roberta Calabrese, Michele Zampieri, Maria Giulia Bacalini, Massimo De Felici, Paola Caiafa

**Affiliations:** 1 Department of Cellular Biotechnologies and Hematology, Sapienza University of Rome, Rome, Italy; 2 Pasteur Institute-Fondazione Cenci Bolognetti, Rome, Italy; 3 Department of Public Health and Cell Biology, University of Rome Tor Vergata, Rome, Italy; 4 Department of Anatomy, Histology, Forensic Medicine and Orthopedics, Sapienza University of Rome, Rome, Italy; University of Bonn, Institut of Experimental Hematolgy and Transfusion Medicine, Germany

## Abstract

Poly(ADP-ribosyl)ation regulates chromatin structure and transcription driving epigenetic events. In particular, Parp1 is able to directly influence DNA methylation patterns controlling transcription and activity of Dnmt1. Here, we show that ADP-ribose polymer levels and Parp1 expression are noticeably high in mouse primordial germ cells (PGCs) when the bulk of DNA demethylation occurs during germline epigenetic reprogramming in the embryo. Notably, Parp1 activity is stimulated in PGCs even before its participation in the DNA damage response associated with active DNA demethylation. We demonstrate that PARP inhibition impairs both genome-wide and locus-specific DNA methylation erasure in PGCs. Moreover, we evidence that impairment of PARP activity causes a significant reduction of expression of the gene coding for Tet1 hydroxylases involved in active DNA demethylation. Taken together these results demonstrate new and adjuvant roles of poly(ADP-ribosyl)ation during germline DNA demethylation and suggest its possible more general involvement in genome reprogramming.

## Introduction

Epigenetics plays a crucial role in regulating cell lineage determination through the action of DNA methylation and chromatin remodeling machineries. Thus, the capability of a cell to restore the omnipotence of DNA needs extensive resetting of genome in order to erase hallmarks defined by epigenetic modifications [1], [2]. During mammalian development, genome-wide epigenetic reprogramming takes place in preimplantation embryo [3] and in primordial germ cells (PGCs), the embryonic precursors of gametes [4]–[6]. Soon after fertilization, the genome of paternal pronucleus is actively demethylated through a DNA repair-driven process as consequence of 5-methylcytosine (5meC) to 5-hydroxymethylcytosine (5hmeC) conversion [7], [8]. Maternal genome is instead demethylated passively during the subsequent embryonic divisions [3]. In germline, overall chromatin changes mostly occur after PGC arrival into the gonadal ridges at the embryonic day 11.5 (E11.5) [6], [9], [10] and they involve histone modifications and a well-characterized widespread DNA demethylation [4]–[6]. Such events are essential for generation of totipotent gametes with proper sex-specific imprints and for erasure of epimutations which may lead to inheritance of disease phenotypes [1], [11]. PGC epigenetic reprogramming resembles the active DNA demethylation process of paternal pronucleus involving the base excision repair (BER) machinery [2], [11], [12]. BER response in PGCs, possibly initiated by thymine DNA glycosylase (Tdg) activity [13], may follow 5meC deamination as well as 5meC hydroxylation [9], [11]. Activation-induced (cytidine) deaminase (Aid) actively participates in PGC DNA demethylation [14] and a strong induction of *ten-eleven translocation 1* (*Tet1*) gene, encoding one of the enzymes that convert 5meC to 5hmeC [15], has been shown in E11.5 PGCs [11], [12].

Although paternal pronucleus and PGCs seem to share similar active demethylating mechanisms [11], [12], the outcome of these processes is quite different on imprinted loci [2], [16]. In fact, the activity of DNA methyltransferase 1 (Dnmt1) preserves methylation state of differentially methylated regions (DMRs) of imprinted genes in parental pronuclei [17], [18], while DMRs are demethylated in PGCs even though this enzyme is continuously expressed during DNA demethylation [6], [19]. Notably, no inhibitory mechanisms of Dnmt1 activity have been revealed during PGC genome resetting.

Considering the pleiotropic functions of poly(ADP-ribosyl)ation (PARylation) in the regulation of epigenetic events [20]–[22], the present study aims to investigate the involvement of this enzymatic reaction in the extensive epigenetic reprogramming occurring in mouse PGCs from E10.5 to E13.5. PARylation is a post-translational modification catalyzed by the poly(ADP-ribose) polymerase (PARP) family enzymes which generate highly electronegative biopolymer transferring ADP-ribose moieties from NAD^+^ to glutamate, aspartate or lysine residues [23] of acceptor proteins (heteromodification) or to PARPs themselves (automodification) [23]–[25]. Moreover, non-covalent interaction with protein-bound or free ADP-ribose polymers (PARs) can also occur [23], [26], [27]. PARylation modifies proteins belonging to different pathways: histones, chromatin enzymes, transcription factors and components of DNA damage response [23], [28]–[32]. Covalent and non-covalent PARylation of target proteins can alter their functionality through both steric and charge effects, affecting protein-protein or protein-DNA interactions, enzymatic activity, or subcellular localization [23]. PARylation is made reversible by the action of the poly(ADP-ribose) glycohydrolase (Parg) which is able to hydrolyze PARs [33]. The founding member of the PARP family is Parp1 [25]. Generally, its enzymatic activity is low in unstimulated cells with PAR half-life of several hours [34]. However, Parp1 activity is dramatically activated by DNA lesions [35], [36], hairpin and cruciform DNA regions [37] as well as various protein partners [38]–[40]. Parp1 is able to control DNA methylation patterns [20], [22] through a combined regulation of Dnmt1 expression [41] and activity [42]. Automodified isoforms of Parp1 localize on and protect the unmethylated state of CpG-rich regions by the inhibition of Dnmt1 activity [41], [43], [44]. Ablation of PARylation restores Dnmt1 function inducing hypermethylation of Parp1-protected loci [41], [43], [44]. On the contrary, hyperactivation of Parp1 is directly able to induce widespread DNA hypomethylation due to a continuous inhibition of Dnmt1 [22], [40]. Furthermore, Parp1 orchestrates chromatin dynamics acting on histones and chromatin remodeling enzymes [30], [31]. In all these ways Parp1 can modulate epigenome and transcription [20], [45].

However, Parp1 is typically known for its involvement in cellular responses to a broad spectrum of DNA damage acting as molecular sensor of DNA lesions. The recognition of both single strand breaks (SSBs) or double strand breaks (DSBs) induces Parp1 auto- and heteromodification reactions for the recruitment of downstream DNA repair effectors [20], [25]. Notably, also Parp2, which accounts for remaining and sufficient PARP activity in absence of Parp1 [46], is involved in DNA damage repair pathways [28].

In the light of the several roles played by PARylation, in the present work, we investigated its participation in DNA demethylation of mouse PGCs in addition to BER response.

## Results

### PGCs Show High PAR Levels before and During Genome-Wide DNA Demethylation

**Figure 1:**
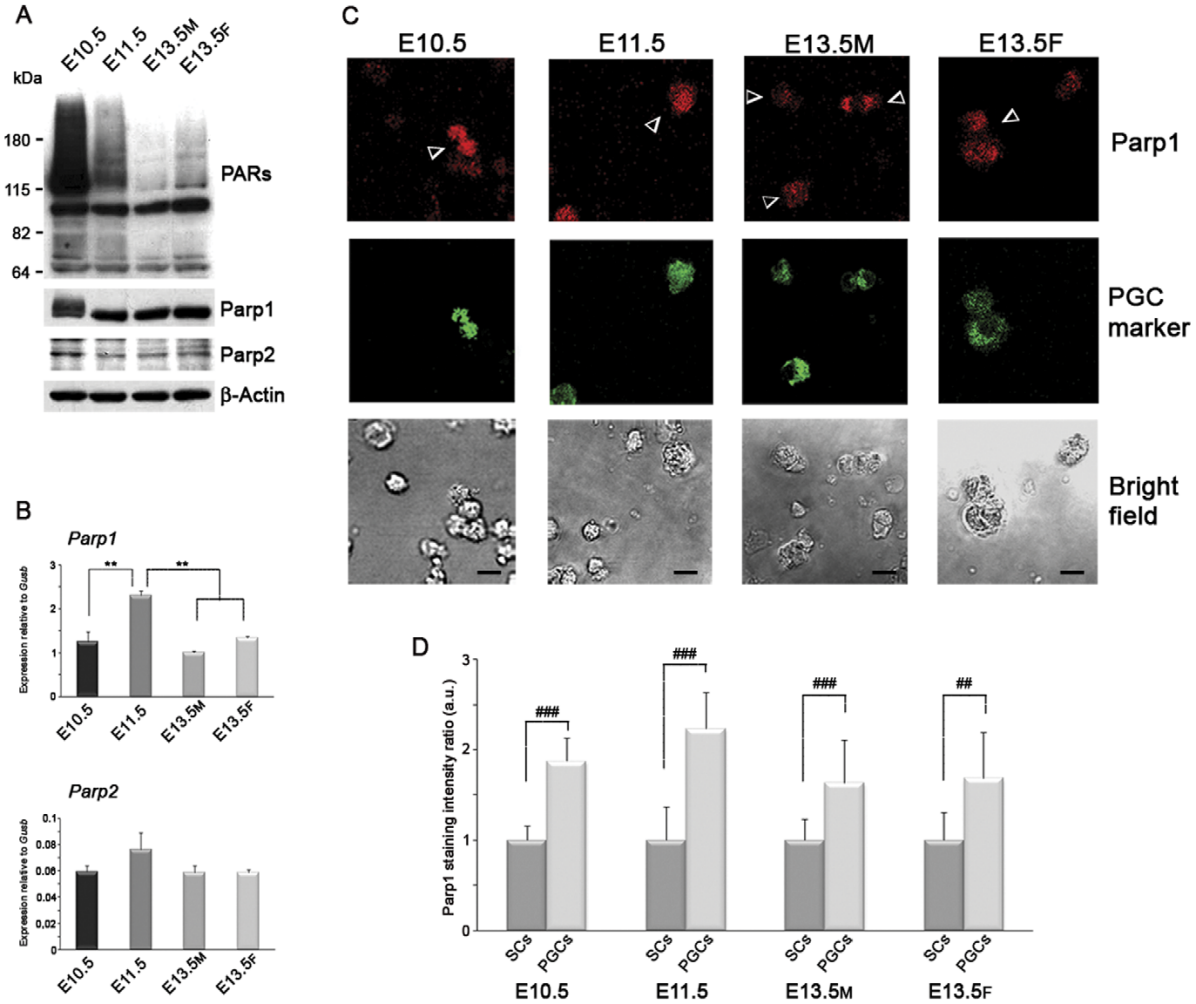
PGCs Show High PAR Synthesis Dependent on Parp1 Before and at the Beginning of Genome Demethylation. (A) Western blot analysis performed on purified PGCs showing high PAR levels in E10.5–E11.5 PGCs and the expression of Parp1 and Parp2 enzymes. (B) qRT-PCR analysis on purified PGCs of *Parp1* and *Parp2* gene expression (mean±s.d., n = 3). Statistically significant differences were obtained using One-way ANOVA test followed by Tukey post test (**p<0.01). (C) Immunofluorescence analysis of Parp1 in PGCs and neighboring SCs. Arrowheads indicate PGCs. Scale bar, 10 µm. (D) Quantification of Parp1 staining in PGCs shown as a ratio between 5meC signal from PGCs relative to the signal from SCs at each developmental stage. Marked differences of Parp1 levels between PGCs and neighboring SCs were observed (mean±s.d). Statistical analysis in PGCs at different developmental stages was performed using Kruskal-Wallis test followed by Dunns post test. Mann-Whitney test was performed to compare PGCs and surrounding SCs at each developmental stage (^##^p<0.01; ^###^p<0.001). a.u., arbitrary unit. M, male. F, female.

The involvement of Parps and PARylation in DNA demethylation during germline establishment was investigated in PGCs purified from CD-1 mice. These cells exhibited progressively diminishing levels of 5meC from E10.5 to E13.5 (Figure S1) and therefore the same timing of DNA demethylation observed in mice with different genetic backgrounds [6], [10], [14]. PAR levels were determined by western blotting analysis on E10.5, E11.5 and E13.5 PGCs and surrounding somatic cells (SCs) as a control. E10.5 PGCs, in which the bulk of DNA demethylation has not yet occurred, showed the highest amount of PARs (Figure 1A). High PAR levels were still detectable in E11.5 PGCs when major DNA demethylation starts while they decreased at the end of the demethylation process both in male and female E13.5 PGCs (Figure 1A). Anti-PAR signal in E10.5 and E11.5 PGCs was mostly predominant at high protein molecular weights shifting up from the molecular weight of Parp1 (116 kDa) (Figure 1A). This is indicative of a massive presence of the automodified isoforms of Parp1 and/or other covalently PARylated proteins of high molecular weight. Of the two highly homologous Parp enzymes [25], Parp1 and Parp2, the former showed more abundant expression both at protein and mRNA levels at all stages examined (Figure 1A, B). The immunoblotting with anti-Parp1 antibody showed a peculiar smear in E10.5 PGCs, confirming the presence of high amounts of automodified Parp1 (Figure 1A). This automodification of Parp1 makes difficult a precise comparison of Parp1 levels between E10.5 and E11.5 by western blot. In fact, qRT-PCR evidenced an up-regulation of *Parp1* transcript at E11.5 (Figure 1B) and further analysis of Parp1 protein in single cells performed by confocal microscopy confirmed a mild increase (Figure 1C, D). In SCs, PAR levels were almost undetectable at E10.5 and variable at later stages while the expression of Parp1 and Parp2 was always significantly lower than in PGCs (Figure S2A, B).

### Parp1 Activation in PGCs is Likely to be Independent of DNA Damage

**Figure 2:**
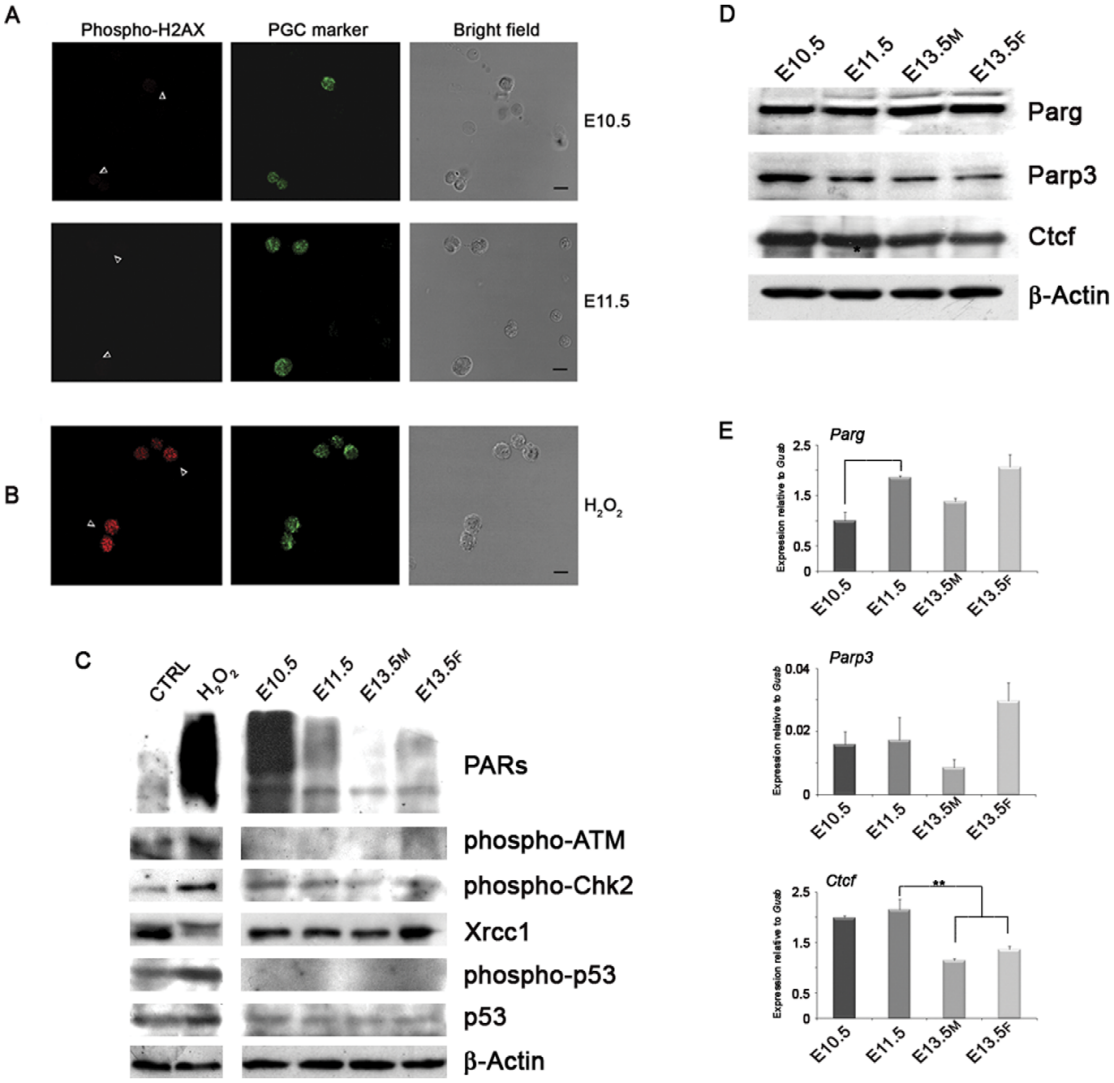
Analyses of Factors Able to Activate PARylation in PGCs. (A) Immunofluorescence analyses of DNA break marker phospho-H2AX did not reveal any positive staining in E10.5–E11.5 PGCs. (B) E11.5 PGCs treated for 5 min with 1 mM H_2_O_2_ were used as positive control for phospho-H2AX staining. Arrowheads indicate PGCs. Scale bar, 10 µm. (C) Western blot analysis on PGCs of proteins involved in DNA damage response showing the presence of phospho-Chk2 and Xrcc1 at all stages. CTRL and H_2_O_2_ represent mouse L929 cells untreated or treated with 1 mM H_2_O_2_ for 10 min, respectively. (D) Western blot analysis performed on purified PGCs showing nearly constant expression of Parg protein. A mild increase of Parp3 and Ctcf protein levels was detected in E10.5 PGCs. (E) qRT-PCR analysis carried out on purified PGCs (mean±s.d., n = 3). Statistically significant differences were determined by One-way ANOVA test followed by Tukey post test (*p<0.05; **p<0.01). M, male. F, female.

Several events including SSBs and DSBs may trigger PARP activation and the consequent PARylation [20], [25]. We checked for the activation of DNA damage pathways in PGCs using antibodies against the phosphorylated isoforms of histone H2AX, ataxia telangiectasia-mutated (ATM), checkpoint kinase 2 (Chk2) and p53 (Figure 2A, C). Only phospho-Chk2 was detected but at all PGC stages. Furthermore, we also analysed the BER component X-ray repair cross-complementing protein 1 (Xrcc1), which was always abundantly expressed in PGCs (Figure 2C).

The analysis of Parg expression, the primary enzyme responsible for PAR catabolism [33], suggested that the high PAR content in PGCs did not depend on its down-regulated levels. In fact, we found that Parg protein did not vary in PGCs during the analysed stages and that its transcripts even increased between E10.5–E11.5 (Figures 2D, E and S3A). Since Parp1 can be activated by interaction with protein partners such as Parp3 and CCCTC-binding factor (Ctcf) [38], [40], we evaluated the expression of these proteins. Despite no detectable differences in *Parp3* transcription (Figure 2E), a slightly higher protein level was observed in E10.5 PGCs (Figures 2D and S3B). Interestingly, Ctcf expression was higher both at protein and mRNA levels in E10.5 and E11.5 PGCs than in E13.5 PGCs (Figures 2D, E and S3C).

### Inhibition of PARylation Impairs Locus-Specific and Global DNA Demethylation in PGCs

In order to elucidate the role of PARylation in PGC DNA demethylation process, we used 3-aminobenzamide (3AB), a competitive inhibitor of PARP activity. Organ cultures of aorta-gonad-mesonephros regions (AGMs) isolated from E10.5 embryos containing migratory and gonadal PGCs were performed.

The levels of PARs were significantly decreased in explants cultured for 72 hrs in the presence of 3AB in comparison to controls (CTRL), indicating an efficient inhibition of PARP activity (Figure S4A). No evident effect of 3AB on cell survival (according to trypan blue assay and count of alkaline phosphatase positive PGCs) was observed (data not shown). Moreover, both CTRL and 8 mM 3AB-treated PGCs were positive for proliferating cell nuclear antigen (Pcna) (Figure S4B), a cell proliferation marker [47].

As expected, *DEAD(Asp-Glu-Ala-Asp) box polypeptide 4* (*Ddx4*) (also known as *Mvh*) and *synaptonemal complex protein 3* (*Sycp3*) genes, whose expression is specifically activated in E11.5 PGCs (Figure S4C) [48], were normally induced in CTRL PGCs in our culture conditions (Figure S4D). Notably, treatment with both 14 mM and 8 mM 3AB markedly impaired their expression (Figure S4D).

**Figure 3:**
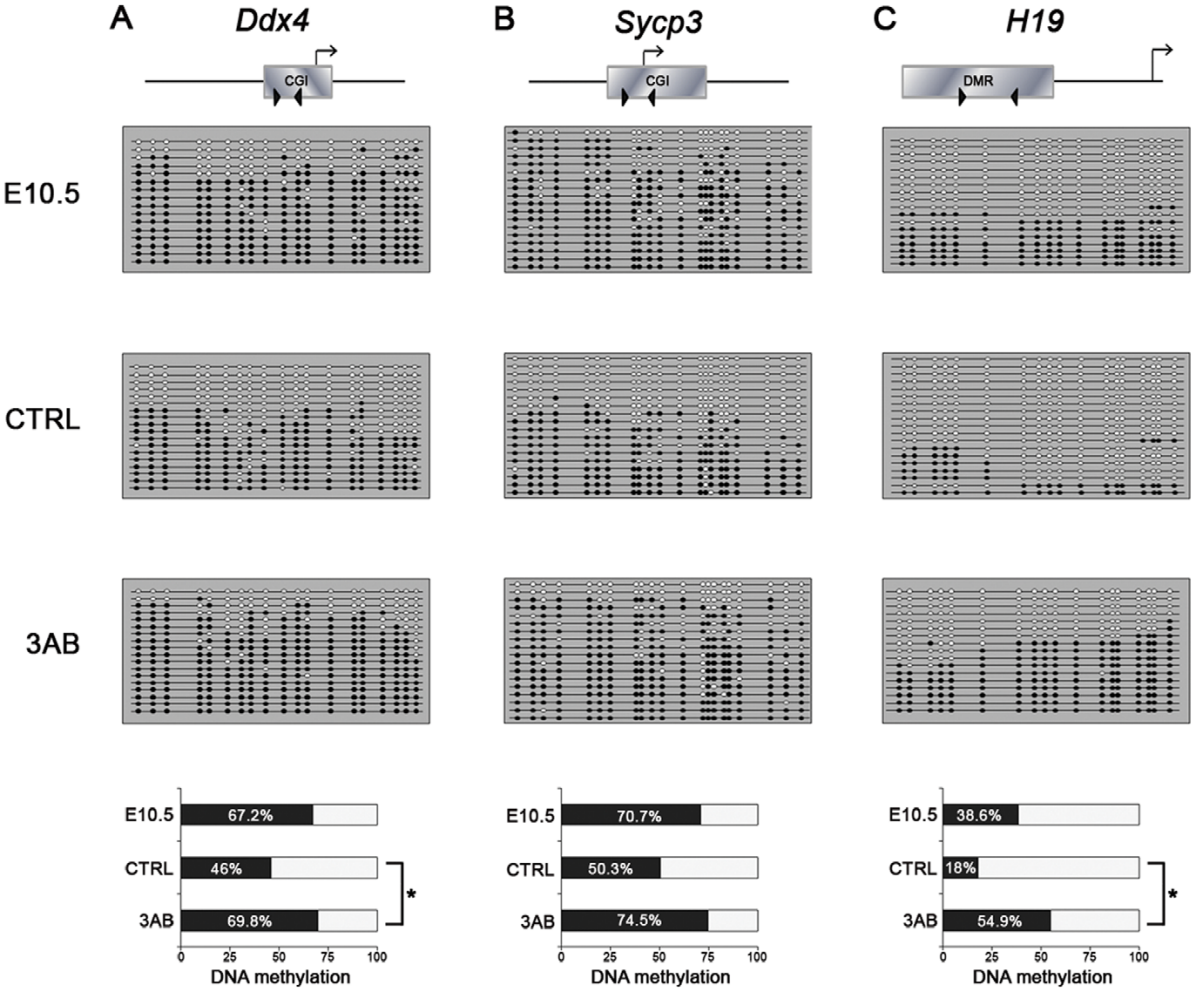
PARP Inhibition Affects DNA demethylation of PGCs at Specific Loci. (A-C) Bisulfite sequencing analyses of DNA methylation of *Ddx4* and *Sycp*3 CGIs and *H19* DMR were performed on PGCs purified from cultured E10.5 AGMs for 72 hrs (CTRL = Control and 3AB). 8 mM 3AB was used for treatment. PGCs purified from E10.5 AGMs corresponding to the starting point of treatment and prior to the beginning of DNA demethylation are identified by E10.5. Each line represents a unique DNA clone; filled and open circles represent methylated and unmethylated CpGs, respectively. Histograms represent the percentage of methylated CpGs. A schematic illustration of the analysed genes is shown above bisulfite result representations. Arrowheads define the region sequenced, while arrows define the transcription start site of the genes. Statistically significant differences were determined by Kruskal-Wallis test followed by Dunns post test (*p<0.05).

Since the activation of these genes depends on the DNA demethylation of their CpG islands (CGIs) [48], DNA methylation analyses of such CGIs were performed after 3AB treatment by bisulfite sequencing method. While CTRL PGCs underwent marked *Sycp3* and *Ddx4* CGI demethylation, no substantial change was observed in PGCs cultured in the presence of 3AB in comparison to E10.5 PGCs (Figure 3A, B). These results were in agreement with the reduced transcription of these genes after PARP inhibition (Figure S4D). Likewise, PGCs were also unable to efficiently erase methylation at the imprinted loci *Igf2/H19* and *Peg3* in presence of 3AB (Figures 3C and S5).

**Figure 4:**
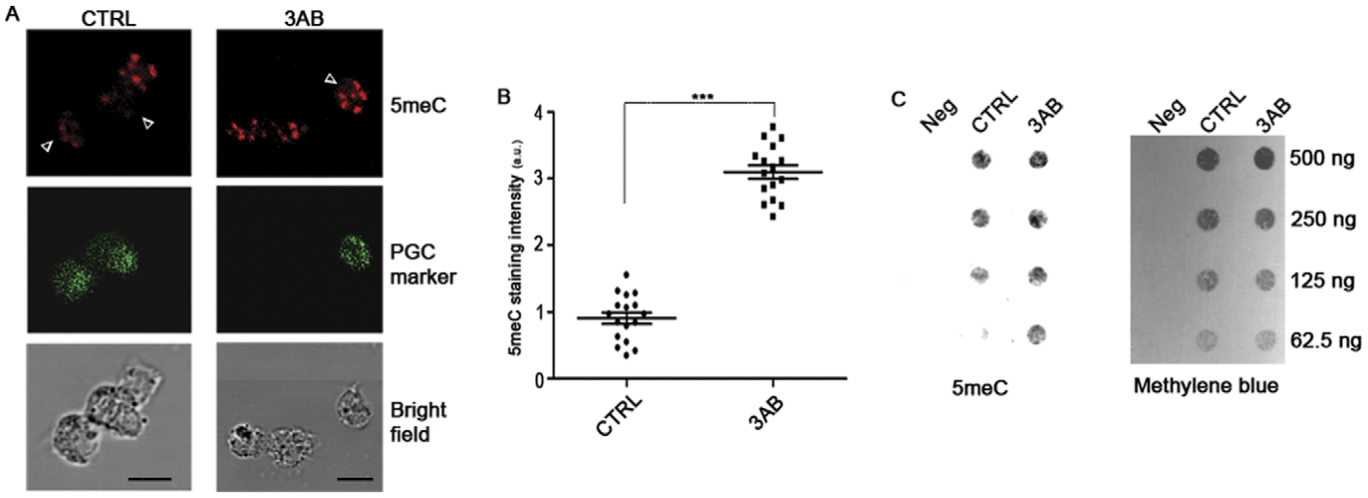
PARP Inhibition Preserves Global Level of 5meC in PGCs. (A) Representative images of control and 8 mM 3AB-treated cells cultured for 72 hrs. Arrowheads indicate PGCs. Scale bar, 10 µm. (B) Quantification of 5meC staining evidencing that PAR-depleted PGCs maintained higher level of global 5meC than control PGCs after 72 hrs of culture (mean±s.e.m.). Statistically significant differences were determined by Mann-Whitney test (***p<0.001). a.u. =  arbitrary unit. (C) Dot-blot assay performed on DNA from CTRL and 8 mM 3AB-treated PGCs using anti-5meC antibody. Neg  =  negative control.

Since bisulfite sequencing method does not discriminate between 5meC and 5hmeC [49], confirmatory analyses of 5meC levels were performed by immunofluorescence and dot-blot assay with anti-5meC antibody. Quantification of 5meC fluorescence and dot-blot results clearly revealed that cultured PGCs treated with 3AB for 72 hrs preserved higher levels of DNA methylation than CTRL (Figures 4A–C and S6). Moreover, PGCs cultured with/without 3AB for 48 hrs already showed the same trend (Figure S7). The use of additional inhibitors of PARP activity as ABT-888 and PJ-34 confirmed the evidence that global DNA methylation was maintained in PGCs when PARP activity was affected (Figure S8).

### Parp1 Acts on PGC Genome Demethylation before BER Activation

**Figure 5:**
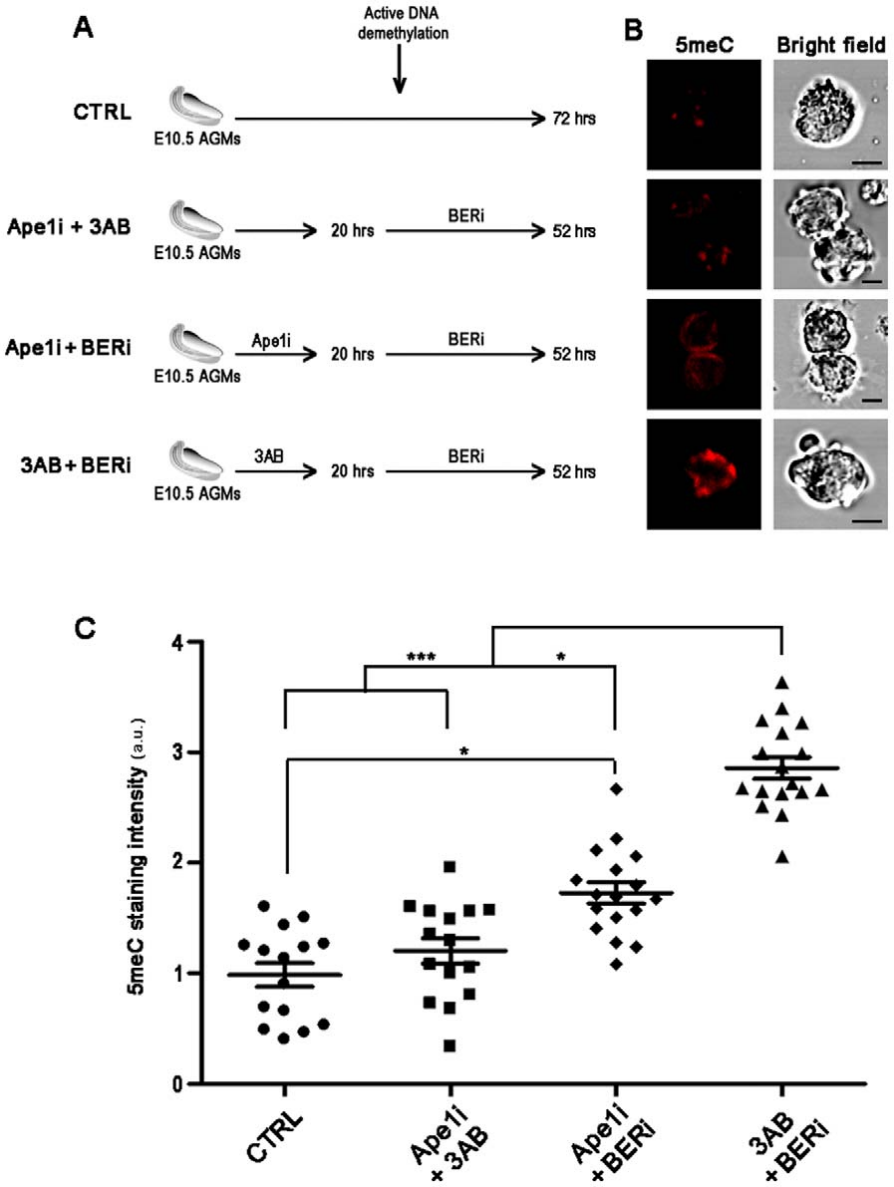
PARP Activation in E10.5 PGCs Has a Role in the DNA Demethylation Process. (A) Schematic representation of treatments performed on E10.5 AGMs with PARP inhibitor 3AB and Ape1 inhibitor CRT4400876 (Ape1i). BERi indicates co-treatment of AGMs after 20 hrs of culture with both 3AB and CRT4400876 for 52 hrs. (B) Immunofluorescence analyses with anti-5meC antibody of AGMs-purified PGCs treated as shown in panel A. Scale bar, 10 µm. (C) Quantification of 5meC staining demonstrating that pre-treatment of PGCs with 3AB before inhibition of BER response efficiently impaired DNA demethylation with respect to all other conditions (mean±s.e.m.). Statistically significant differences were determined by Kruskal-Wallis test followed by Dunns post test (*p<0.05; ***p<0.001). a.u. =  arbitrary unit.

It has been postulated that active DNA demethylation in PGCs occurs between E11.5–E12.5 as a BER-dependent process and that PARP activation can be caused by DNA SSBs generated in the context of BER response [12]. However, we observed that PARylation is noticeably high in E10.5 PGCs (Figure 1A) before the beginning of DNA demethylation process at E11.5 and that markers of DNA damage and BER are absent in PGCs or expressed independently of the bulk of DNA demethylation (Figure 2A, C). To investigate the effective roles of PARylation before BER activation, E10.5 AGMs were treated with PARP inhibitor 3AB and/or CRT0044876 (Ape1i), a specific inhibitor of the apurinic-apyrimidinic endonuclease 1 (Ape1) which is the major abasic endonuclease involved in BER [50] (Figure 5A). Global DNA methylation changes in PGCs were checked by quantitative analyses of 5meC fluorescence. PGCs treated with Ape1i alone from the beginning of culture showed low levels of 5meC like CTRL (data not shown). Similarly, low 5meC staining was also observed when Ape1i and 3AB were added together to E10.5 AGMs after 20 hrs of culture (Ape1i +3AB), developmental stage in culture comparable to E11.5 (Figure 5B, C). This condition was performed to impair BER response completely by inhibiting both Ape1 and Parp1 actions. On the contrary, PGCs cultured for 20 hrs in presence of Ape1i or 3AB followed by the addition of both inhibitors together (Ape1i or 3AB + BERi), showed significant or very significant higher level of DNA methylation, respectively, in comparison to CTRL (Figure 5B, C).

### Expression of Components of DNA Methylation Machinery in PGCs

Dnmt1 is the main DNA methyltransferase expressed in PGCs and it is still localized in their nucleus during the period of DNA demethylation. Dnmt1 exerts both maintenance and de novo DNA methylation activity [51], [52] and mechanisms able to prevent such actions in PGCs should exist. On the basis of our previous results showing that Parp1 is able to inhibit Dnmt1 activity [22], we hypothesized that high PAR levels in PGCs may also play an inhibitory effect on Dnmt1. Since a direct analysis of Dnmt1 activity on the little number of available PGCs resulted unfeasible, we verified the availability of DNA methylation machinery components in PGCs. In particular, we evaluated the expression of Dnmt enzymes in PGCs and of co-factors known to recruit Dnmt1 onto DNA or to regulate its activity such as ubiquitin-like-containing PHD and ring finger domain 1 (Uhrf1) [51], Pcna [53], [54] and DNA methyltransferase associated protein 1 (Dmap1) [55] in relation to PARP activity dynamics.

**Figure 6:**
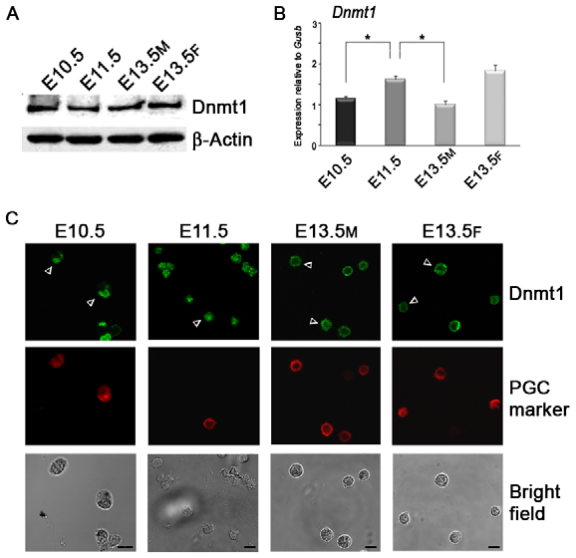
Maintenance Dnmt1 Enzyme Is Continuously Expressed in PGCs Undergoing DNA Demethylation. (A) Western blot analysis performed on purified PGCs showing constant expression of Dnmt1 protein. (B) qRT-PCR analysis of *Dnmt1* gene expression carried out on purified PGCs (mean±s.d., n = 3). Statistically significant differences were determined by One-way ANOVA test followed by Tukey post test (*p<0.05). (C) Immunofluorescence analysis evidenced nuclear staining of Dnmt1 in E10.5–E11.5 PGCs and its peripheral localization in E13.5 PGCs. Arrowheads indicate PGCs. Scale bar, 10 µm. M, male. F, female.

We confirmed that Dnmt1 was expressed in PGCs at mRNA and protein levels (Figure 6A, B). Although higher levels of transcripts were observed in E11.5 PGCs and E13.5 female PGCs (Figure 6B), the amount of Dnmt1 protein did not change during the period of DNA methylation resetting (Figures 6A and S9A) indicating that probably it is its activity that is affected. Immunofluorescence analysis also confirmed the nuclear localization of Dnmt1 in E10.5–E11.5 PGCs (Figure 6C). Surprisingly, we observed that around E13.5, when PAR levels are significantly reduced (Figure 1A), Dnmt1 translocated progressively from the nucleus to the cytoplasm in most of male and female PGCs (Figures 6C and S9B). The latter result was confirmed by biochemical analyses (Figure S9C). We also confirmed that *Dnmt3a* and *Dnmt3b* were expressed at mRNA levels in PGCs at all stages examined and nearly at constant levels (Figure S10) [56]. However, Dnmt3a protein was absent in PGCs and Dnmt3b was localized in the cytoplasm [6]. A peculiar profile was evidenced for *Dnmt3l* gene expression, which showed a clear peak in male E13.5 PGCs (Figure S10).

**Figure 7:**
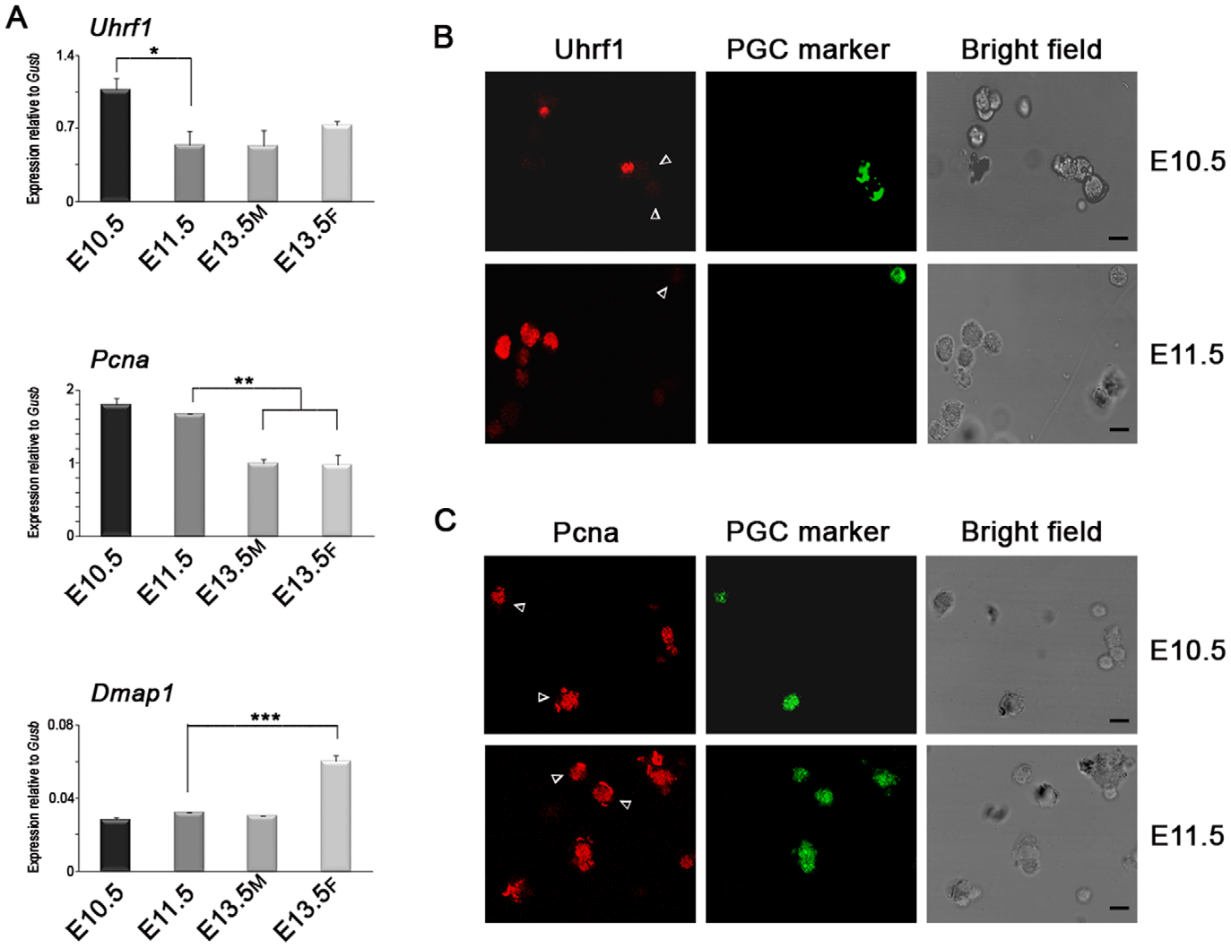
Expression of Dnmt1 Co-factors Does Not Change in PGCs When DNA Demethylation Starts. (A) qRT-PCR analysis of *Uhrf1*, *Pcna* and *Dmap1* performed on purified PGCs (mean±s.d., n = 3). Statistically significant differences were determined by One-way ANOVA test followed by Tukey post test (*p<0.05; **p<0.01; ***p<0.001). (B) Immunofluorescence analysis with anti-Uhrf1 antibody evidenced that this enzyme was never present in PGCs prior to and at the beginning of the epigenetic reprogramming. (C) Immunofluorescence analysis against Pcna demonstrated its expression in PGCs both at E10.5 and E11.5. Arrowheads depict PGCs. Scale bar, 10 µm. M, male. F, female.

Our analyses of Dnmt1 co-factors showed that *Uhrf1* gene, previously reported to be down-regulated during PGC specification by B-lymphocyte-induced maturation protein 1 (Blimp1) [57], was actually expressed at mRNA level in E10.5 PGCs and down-regulated at later stages (Figure 7A). However, the protein was not detectable by immunofluorescence in PGCs while it was expressed in a subset of neighboring SCs (Figure 7B). *Pcna* expression at mRNA level was high at E10.5 and E11.5 and progressively down-regulated in both genders at later stages while *Dmap1* transcription was low at these stages and up-regulated in female E13.5 PGCs (Figure 7A). Pcna was detected in the nucleus of PGCs and SCs at all stages examined (Figure 7C).

### PARylation Modulates the Transcription of *Tet1* Gene

The maintenance of high 5meC staining observed after PAR depletion (Figures 4 and S8) might also depend on the transcriptional regulation mediated by PARylation [20] of genes involved in the control DNA methylation state in PGCs. For this reason, we evaluated the expression of *Dnmt1*, *Pcna* and *Parp1*. However, the transcription of none of these genes was affected by 3AB treatment (Figure S11A) as well as protein stability of DNA methylation machinery components (Figures S4B and S11B).

**Figure 8:**
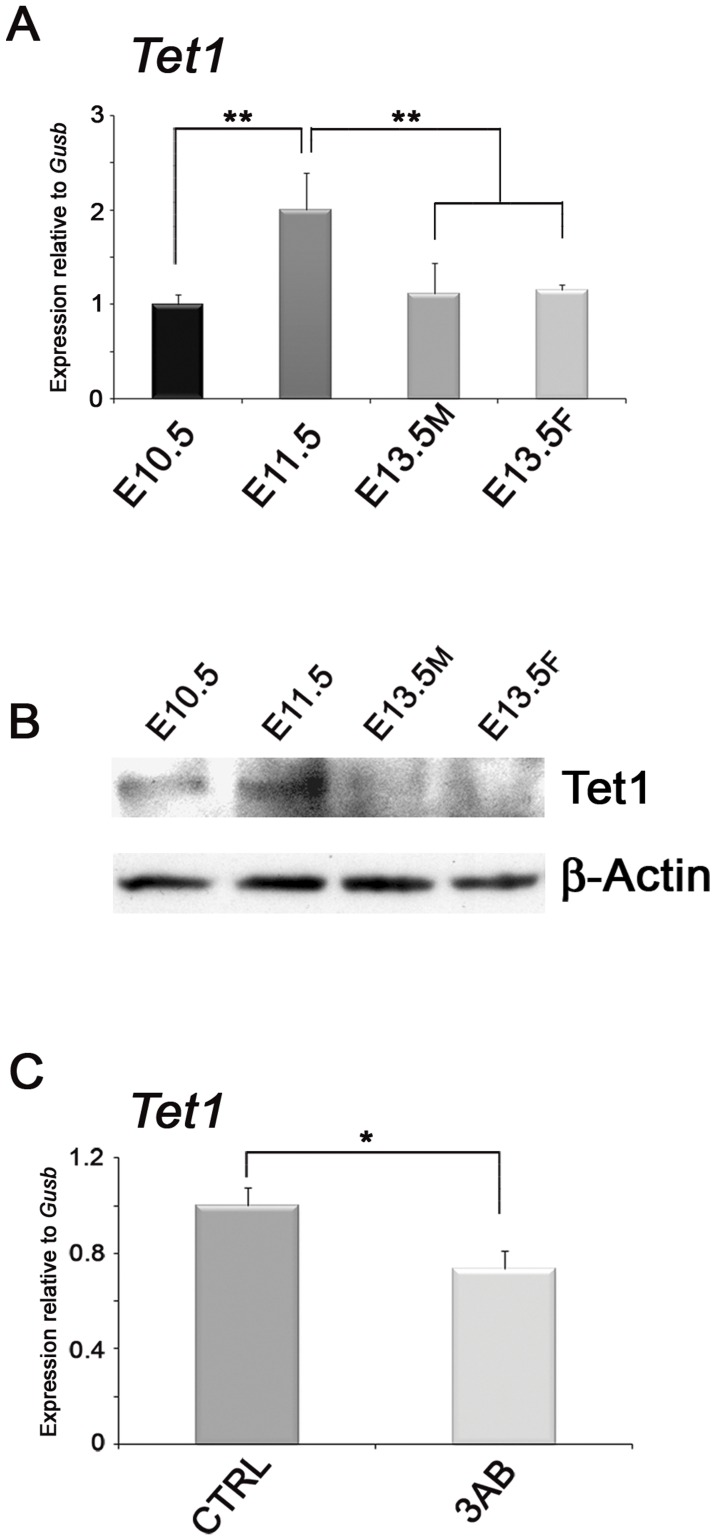
PARP Inhibition Affects the Expression *Tet1* Gene in PGCs. (A) qRT-PCR analysis of *Tet1* gene carried out on purified PGCs at different developmental stages (mean±s.d., n = 3). Statistically significant differences were determined by One-way ANOVA test followed by Tukey post test (**p<0.01). (B) Western blot analyses of Tet1 protein on PGCs showing the up-regulation of the enzyme in E11.5 stage. (C) Expression analysis of *Tet1* gene performed by qRT-PCR on PGCs purified from AGMs cultured for 72 hrs with/without 3AB. (mean±s.d., n = 3). Statistically significant differences were determined by paired Student’s t-test (*p<0.05). M, male. F, female.

Therefore, we focused on genes coding for factors reported as being involved in the active DNA demethylation process of PGCs such as *Aicda* (coding for Aid protein) and *Tet* genes. While *Aicda* was not expressed at detectable levels (data not shown), distinct changes in *Tet* gene expression were found in E10.5–E13.5 PGCs (Figures 8A and S12A). In particular, *Tet1* gene was highly expressed in PGCs and an interesting peak of transcription was observed in E11.5 PGCs, which even corresponded to a relevant increase of Tet1 protein level (Figure 8A, B). It is noteworthy that the expression of *Tet1* gene was significantly impaired in PGCs after PARP inhibition (Figure 8C). The analyses of *Tet2* expression revealed a significant increase in female E13.5 PGCs while *Tet3* transcripts were gradually decreased (Figure S12A). Higher levels of *Tet3* were detected in 3AB-treated PGCs while the expression of *Tet2* showed no significant difference (Figure S12B).

## Discussion

In the present paper, we have examined the role of PARylation in mouse PGCs before and during the bulk of DNA demethylation occurring between E10.5–E13.5 [4]. Deamination of 5meC by Aid [14] or hydroxylation of 5meC mediated by Tet enzymes [12] followed by glycosylase-mediated BER have been proposed as the processes governing active DNA demethylation in mouse PGCs [11]. In these models, Parp1 has been proposed as being activated by SSBs and to participate in BER pathway during PGC epigenetic reprogramming [11], [12]. However, PARylation promotes epigenetic changes in several ways also through a direct control of DNA methylation patterns [20]–[22]. This evidence prompted us to investigate if PARP activity might also exert additional roles in genome demethylation of mouse PGCs.

Firstly, we analysed PAR content in PGCs showing that the highest PAR levels were present in E10.5 PGCs thus before the beginning of the bulk of DNA demethylation. The smears revealed by anti-Parp1 antibody and starting from Parp1 molecular weight in anti-PAR immunoblotting suggested a massive presence of the automodified isoforms of Parp1 enzyme in E10.5 PGCs. We observed that PAR synthesis remained high in E11.5 PGCs, as previously reported by immunofluorescence analysis [12], and decreased in E13.5 PGCs when the process is completed. The huge amount of PARs in E10.5 PGCs did not appear to depend on increased levels of Parp1 protein or on absence of Parg expression, which remained unchanged during the period analysed. Furthermore, the elevated level of PARs in PGCs in comparison to surrounding somatic cells argued for a specific role of PARylation during this period. The presence of marks of DNA damage as activators of PARP activity was assessed. Phospho-H2AX is generally used as a marker of DSBs but it can also recognize SSBs as shown in the context of BER-mediated active demethylation during paternal pronucleus reprogramming [58]. However, we detected no phospho-H2AX staining in E10.5–E11.5 PGCs. Similarly, we did not find detectable staining for other two markers of DNA damage such as phosphorylated ATM and p53. We also searched for the active phosphorylated isoform of Chk2 because this enzyme can take part in BER response driving the phosphorylation of scaffold protein Xrcc1 [59]. In this regard, it has been shown that chromatin-bound Xrcc1 was specifically observed in E11.5 PGCs [12]. Using western blotting, we found that phosphorylated Chk2 and Xrcc1 protein were actually present in PGCs but at all stages analysed irrespective of the DNA demethylation period. On the other hand, two Parp1 protein partners able to induce its activation, Parp3 and Ctcf, [38], [40], were slightly up-regulated in E10.5 PGCs. Therefore, we believe that interactions with protein partners and/or other unidentified trans-activating events could also participate in PARP activation but independently of DNA damage.

We obtained strong evidence supporting a crucial role of PARylation for locus-specific and global DNA demethylation during PGC epigenetic reprogramming. In this regard, we demonstrated that inhibition of PARP activity in PGCs with 3AB impaired the DNA demethylation process of two postmigratory germ cell-specific genes, *Sycp3* and *Ddx4*, and of the imprinted loci *Igf2*/*H19* and *Peg3*. To distinguish between 5meC and 5hmeC, we performed immunofluorescence analyses with anti-5meC antibody [49] after 3AB and two other specific inhibitors of PARP activity such as PJ-34 and ABT-888.

Quantification of anti-5meC fluorescence and dot-blot analysis clearly indicated that PARP inhibition impairs DNA demethylation in PGCs. As BER pathway is involved in active DNA demethylation [12] and PARylation participates in BER response [35], [60], we investigated the combined effect of 3AB and CRT4400876, an inhibitor of the Ape1 BER enzyme, on this process. It is to be considered that during BER response Ape1 acts after the removal of atypical bases by DNA glycosylases [50] and thus, it follows the removal of modified 5meC in the context of active DNA demethylation [11]. In fact, treatment with CRT4400876 during BER response causes the persistence of abasic sites on DNA [61]. Consistent with this, simultaneous impairment of Ape1 and PARP activities during the active DNA demethylation (E11.5) showed low levels of 5meC staining. Conversely, a slightly higher 5meC staining was observed when Ape1 inhibition occurred before the beginning of active DNA demethylation. This result can depend on the ability of Ape1 to enhance DNA glycosylase turnover [62], [63] as well as on the complex and controversial interplay between Ape1 and Parp1 [64], [65]. In particular, it has been demonstrated that Parp1 also possesses AP lyase activity capable of incising AP site-containing DNA [65]. As concerns Parp1 action, its activity is triggered by SSBs introduced by Ape1 thus, also in this case, after the removal of 5meC by DNA glycosylases [11]. Therefore, the function of PARylation associated with BER pathway should follow the formation of abasic sites [50]. Notably, we found that E10.5 PGCs pre-treated with PARP inhibitor instead showed significantly higher levels of 5meC compared to all other conditions. All together these results suggest that even if BER-mediated demethylation occurs in PGCs, Parp1 can participate to DNA demethylation with roles independent of BER pathway.

The last series of results reported in the present paper suggest two additional ways by which Parp1 could exert its action on the DNA methylation erasure in PGCs. Firstly, PARylation could favour DNA demethylation in PGCs by inhibiting Dnmt1 activity as demonstrated to occur in mammalian somatic cells [41], [42], [44]. Since PGCs are still dividing between E10.5 and E12.5 [66], [67], PAR-mediated impairment of Dnmt1 maintenance activity can favour passive demethylation and, differently from parental pronuclei after fertilization, imprinting erasure. Notably, Dnmt1 even possesses de novo activity [52], [68], [69], that could affect newly demethylated cytosines, and maintenance activity independent of DNA replication [70]. In particular, this latter occurs throughout G2 cell cycle phase [70], when the main part of DNA demethylation takes place in E11.5 PGCs [5]. Although we were unable to directly measure Dnmt1 activity in PGCs because of the small number of available cells, several observations indicated that Dnmt1 could still be active during the period of maximum DNA demethylation in absence of proper inhibition. In fact, we evidenced that Dnmt1 was expressed at constant protein levels and localized within the nucleus of E10.5–E11.5 PGCs [6], [19]. Moreover, we did not obtain any indication about changes in expression of Dnmt1 co-factors that could prevent its enzyme activity. Other indirect evidence about the possible inhibitory action of PARylation on Dnmt1 was the observation that when PAR levels underwent a marked decrease in E13.5 PGCs, Dnmt1 was progressively translocated into the cytoplasm. Since Dnmt1 preferentially acts on DNA structures associated with recombination [71], its cytosolic translocation might avoid inappropriate methylation of DNA during meiosis, which is about to begin [10]. Furthermore, PGCs treated with Dnmt1 inhibitors or deriving from *Dnmt1* hypomorphic mice showed anticipated expression of germ cell-specific genes normally activated only after DNA demethylation [48], [72]. Also this evidence supports the idea that Dnmt1 retains its activity in these periods and that inhibitory mechanisms as PARylation are necessary.

A second possible mechanism through which PARylation might rule DNA methylation in PGCs is by transcriptional regulation of key genes controlling this process. Expression analyses performed on PGCs purified from 3AB-treated AGMs showed that the transcription of *Dnmt1*, *Pcna* and *Parp1* was not impaired while expression of *Tet* genes was altered. Currently, several papers have demonstrated the relevance of Tet enzymes in the active DNA demethylation processes [73], [74]. In particular, Tet3-mediated hydroxylation of 5meC and the other Tet-mediated modifications, 5-formylcytosine and 5-carboxylcytosine, have been evidenced in the DNA demethylation process of paternal pronucleus at fertilization [7], [8], [75], [76]. During germline reprogramming, protein and transcript levels of Tet1 are specifically up-regulated in E11.5 PGCs strongly supporting the involvement of active DNA demethylation process dependent on 5meC hydroxylation in PGCs [11]. In this context, the ability of PARylation to modulate the transcription of *Tet* genes is relevant. In particular, PARP activity seems necessary for the up-regulation of *Tet1* expression and consequently for the beginning of active demethylation. In addition, 5hmeC itself may induce passive DNA demethylation by excluding Dnmt1 action during DNA replication [2], [77].

In conclusion, our results demonstrate that Parp1 is crucially involved in genome-wide DNA demethylation of PGCs. A plural contribution of PARylation, which is also triggered independently of DNA damage, appears to have a direct role in the extensive reprogramming of germline. Besides participating in BER response, Parp1 activity may favour DNA demethylation in PGCs by inhibiting Dnmt1 activity but, above all, it can initiate active DNA demethylation through the transcriptional up-regulation of *Tet1* gene.

## Materials and Methods

### Gonadal Ridges and PGC Collection

All experiments were carried out in compliance with accepted standard of humane animal care and with the approval of relevant national (Ministry of Welfare) and local (Institutional Animal Care and Use Committee, Tor Vergata University) committees. CD-1 female mice (Charles River, Italy) were mated with CD-1 male mice and the detection of a vaginal plug the morning following mating was designated as E0.5. PGCs were obtained from E10.5–E13.5 CD-1 mice embryos as previously described [78]. Briefly, hindgut, dorsal mesentery and urogenital ridges (E10.5 AGM), urogenital ridges (E11.5) and gonads (E13.5) were dissected from CD-1 mouse embryos. At E13.5 the embryos were sexed by the morphology of the gonads. AGM and gonads were then dissociated in Trypsin/EDTA in a single cell suspension and PGCs were isolated with the use of immunomagnetic cell sorting (Mini Macs, Miltenyi). Alkaline phosphatase expression was used as a marker for PGCs to estimate the number and the percentage of PGCs in the cell suspensions after purification. PGC purity was 75–80% for E10.5 PGCs, 85–95% for E11.5 PGCs, 85–95% for male and female E13.5 PGCs.

### Organ Culture

AGMs were collected from E10.5 embryos and transferred in D-MEM-F12 added with 0.4 mg/ml BSA, 0.25 mM pyruvate, 0.5 mg/ml *N*-acetyl-l-cysteine, (Sigma-Aldrich). When indicated, 3-aminobenzamide (8 or 14 mM) (Sigma-Aldrich), CRT4400876 (100 µM) (Sigma-Aldrich), PJ-34 (3 µM) (Sigma-Aldrich) and ABT-888 (3 µM) (Alexis Biochemicals) dissolved in DMSO were added to the culture medium. DMSO alone was added instead in the controls. Cultures were carried out in constant rotation at 37°C in 5% CO_2_ for the indicated times. For the DNA methylation and gene expression analysis, treated and control PGCs were purified and their viability checked by Trypan blue exclusion test.

### Immunofluorescence Staining

PGCs were spotted onto poly-L-lysine coated slides (Sigma-Aldrich) and fixed with 4% paraformaldehyde for 10 min, washed twice each for 10 min in PBS. The cells were permeabilised for 30 min using PBS, 3% BSA, 0.1% Triton X-100 followed by primary antibody staining in PBS, 3% BSA at 4°C overnight. At the end of incubation the slides were washed in PBS and incubated for 1 hr at room temperature with Alexa fluor conjugated secondary antibodies (Molecular Probes). As regards 5meC staining, PGCs deriving from AGMs cultured with/without 3AB, were permeabilised in PBS, 1% BSA, 0.5% TritonX-100 for 30 min. The slides were then washed with PBS and treated with 4 N HCl for 30 min at 37°C. Following extensive PBS washes, the slides were blocked in PBS, 1% BSA, 0.1% TritonX-100 for 30 min and incubated in the same buffer with anti-5meC antibody at 4°C overnight. The slides were then extensively washed and incubated for 1 hr at RT with Alexa fluor conjugated secondary antibodies, and then treated with RNase A (1 mg/ml) for 30 min. Negative controls were processed in the same condition except primary antibody incubation. Finally, all samples were mounted in buffered glycerol pH 9.5 and immunolocalization was analysed using a Leica confocal microscope (Laser Scanning TCS SP2) equipped with Ar/ArKr and HeNe lasers. The images were scanned under a 40X oil immersion objective. In order to perform a quantitative analysis, mean fluorescence intensity of each fluorophore of interest was calculated using Stack profile quantification tool of Leica confocal software according to manufacturer’s instructions and as previously described [79]. Briefly, spatial series each composed of about 15 optical sections with a step size of 1 µm were performed. Profiles of mean fluorescence intensity were measured within a region of interest (ROI) of equivalent size regions and background values subtracted. Additional quantification of fluorescence intensity was calculated as a ratio between signal recovered from PGCs relative to the average signal from SCs present in each image.

### Western Blot Analysis

Total cell lysates of purified PGCs and somatic cells (SCs) were prepared in RIPA buffer (50 mM Tris-HCl at pH 7.4, 150 mM NaCl, 1% NP-40, 0.5% sodium deoxycholate, 0.1% SDS and 1 mM EDTA) and normalized for protein concentration. Cytosolic fraction was obtained recovering supernatant after centrifugation of purified PGCs previously incubated (15 min in ice) in isolation buffer (10 mM Tris-HCl at pH 7.8, 4 mM MgCl_2_, 1 mM EDTA, 0.5 mM DTT, 1% Triton X-100, 0.25 M Sucrose). Pelleted nuclei were washed once in isolation buffer without Triton X-100, centrifugated and lysated in RIPA buffer. Each buffer was supplemented with protease inhibitor cocktail (complete EDTA-free, Roche Applied Science). Protein extracts were resolved by SDS-PAGE, transferred onto Hybond-ECL nitrocellulose membranes (Amersham Biosciences) and probed with the indicated antibodies.

### Dot-Blot Assay

DNA was denatured in 0.4 M NaOH, 10 mM EDTA at 95°C for 10 min, and then neutralized by adding an equal volume of cold 2 M ammonium acetate (pH 7.0). Next, 2-fold dilutions of denatured DNA samples were spotted on a nitrocellulose membrane Hybond-N^+^ (Amersham Biosciences) in an assembled Bio-Dot apparatus (Bio-Rad). Vacuum was subsequently applied to filter through DNA samples. The blotted membrane was washed with 2X SSC buffer and air-dried. The membrane was then blocked with 5% non-fat milk and incubated with monoclonal 5meC antibody. Binding of an HRP-conjugated secondary antibody was visualized by enhanced chemiluminescence. To ensure equal spotting of total DNA on the membrane, the same blot was then stained with 0.02% methylene blue in 0.3 M sodium acetate (pH 5.2).

### Antibodies

Ssea1 (kind gift of Dr. P. Donovan), PAR (Trevigen), Parp1 (Enzo Life Sciences), Parp2 (Enzo Life Sciences), Parp3 (Enzo Life Sciences), Parg (Santa Cruz), phospho-H2AX (Ser139) (Millipore), Ctcf (Millipore), Dnmt1 (Imgenex), β-Actin (Sigma-Aldrich), α-Tubulin (Sigma-Aldrich), Ddx4 (Abcam), Stella (Abcam), Oct3/4 (Santa Cruz), Uhrf1 (kind gift of Dr. I.M. Bonapace), Pcna (Santa Cruz), 5meC (Eurogentec), phospho-Chk2 (Thr68) (Cell Signalling Technology), phospho-ATM (Ser1981) (Rockland), phospho-p53 (Ser15) (Cell Signalling Technology), Xrcc1 (Abcam), p53 (kind gift of Dr. S. Soddu), Tet1 (Millipore).

### Quantitative Real-time PCR

RNA was extracted with the RNeasy micro kit (Qiagen), and treated with RNase-free DNase (Qiagen). The RNA concentration and purity (260/280 and 260/230 ratios) was analysed using a ND-1000 Spectrophotometer (NanoDrop Technologies). Total RNA was subjected to retrotranscription using SuperScript VILO cDNA Synthesis Kit (Invitrogen). Quantitative PCR reactions were performed with EXPRESS qPCR Supermix Universal (Invitrogen) or SYBR Green Supermix (Bio-Rad) using iCycler IQ detection system (Bio-Rad). Gene expression analysis was performed using the comparative cycle threshold method with *Gusb* for normalization, the most stable reference gene during the analysed developmental stages. It was selected among *Gapdh*, *B2m* and *Hprt* genes using geNORM software [80]. Targets were quantified with Taqman Gene Expression Assays (Applied Biosystems) (Table S1) or specific primer pairs (Table S2).

### Bisulfite Sequencing

DNA was extracted with DNeasy tissue kit (Qiagen) and converted using EZ DNA Methylation Kit (Zymo research). Amplification was performed using specific primer pairs listed in Table 3. Fragment cloning was performed using TOPO TA-cloning vector (Invitrogen) and twenty-five independent clones for each condition were sequenced.

### Statistical Analysis

Statistical tests used for comparison and the number of biological replicates (n) are reported in figure legends. In histograms where different developmental stages are compared, statistically significant differences were represented only between consecutive stages.

## Supporting Information

Figure S1 **DNA Demethylation Dynamics of PGCs Deriving From CD-1 Mouse Embryos.** Immunofluorescence analysis performed with anti-5meC in E10.5–E13.5 PGCs showing gradual loss of DNA methylation. Arrowheads indicate PGCs while arrows indicate SCs. Scale bar, 10 µm.
(TIF)

Figure S2 **Gonadal Somatic Cells Exhibit Different PARylation Profile in Comparison to PGCs.** (A) Western blot analysis performed on control SCs. (B) qRT-PCR analysis carried out on SCs (mean±s.d., n = 3). Unpaired Student’s t-test was performed to compare expression of *Parp1* and *Parp2* between SCs and PGCs in Figure 1B at each developmental stage (#p<0.05; ##p<0.01). M, male. F, female.
(TIF)

Figure S3 **Densitometric Analysis of Parg, Parp3 and Ctcf Protein Expression in PGCs.** (A-C) Blots of Figure 2D were subjected to densitometry analysis using Quantity One software.
(TIF)

Figure S4 **PARP** **Inhibition Affects the Expression of Germ Cell-specific Genes.** (A) Western blot analysis showing ablation of PAR levels in E10.5 AGMs cultured for 72 hrs with 3AB. (B) Immunofluorescence analysis of Pcna expression in control (CTRL) and 8 mM 3AB-treated PGCs. Arrowheads indicate PGCs. Scale bar, 10 µm. (C) qRT-PCR of *Ddx4* and *Sycp3* genes performed on PGCs prior to (E10.5) and at the beginning (E11.5) of DNA demethylation (mean±s.d., n = 3). Statistically significant differences were determined by unpaired Student’s t-test (***p<0.001). (D) Expression analysis of *Ddx4* and *Sycp3* performed by qRT-PCR on PGCs purified from control (CTRL) or 3AB-treated AGMs cultured for 72 hrs. E10.5 identifies PGCs purified from not cultured E10.5 AGMs. (mean±s.d., n = 3). One-way ANOVA test followed by Tukey post test was used to determine statistical differences between CTRL and 3AB-treated PGCs (*p<0.05; **p<0.01) as well as between treated/untreated PGCs with E10.5 PGCs (^#^p<0.05; ^##^p<0.01; ^###^p<0.001).
(TIF)

Figure S5 **PARP Inhibition Affects DNA Demethylation of the Imprinted Locus** ***Peg3.*** Bisulfite sequencing analysis of *Peg3* DNA methylation performed on PGCs purified from cultured E10.5 AGMs for 72 hrs (CTRL = Control and 3AB). 8 mM 3AB was used for treatment. Each line represents a unique DNA clone; filled and open circles represent methylated and unmethylated CpGs, respectively. Histograms represent the percentage of methylated CpGs.
(TIF)

Figure S6 **PARP Inhibition Impairs Global DNA Demethylation.** Quantification of 5meC fluorescence reported in Figure 4B is here shown as a ratio between 5meC signal recovered from PGCs relative to the average signal from SCs (mean±s.e.m.). Statistically significant differences were determined by Mann-Whitney test (***p<0.001). a.u. =  arbitrary unit.
(TIF)

Figure S7 **DNA Methylation Levels in PGCs After 48 hrs of 3AB Treatment.** Quantification of 5meC staining performed on CRTL and 8 mM 3AB-treated PGCs cultured for 48 hrs (mean±s.e.m.) showing the same trend as observed after 72 hrs of culture.
(TIF)

Figure S8 **Specific Inhibitors of PARylation Confirm Data Obtained With 3AB.** (A) Representative images of control (CTRL), ABT-888 and PJ-34-treated PGCs deriving from AGMs cultured for 72 hrs. Scale bar, 10 µm. (B) Quantification of 5meC staining evidencing that additional inhibitors of PARP activity also maintained high levels of global 5meC (mean±s.e.m.). Statistically significant differences were determined by Kruskal-Wallis test followed by Dunns post test (*p<0.05; **p<0.01). a.u. =  arbitrary unit.
(TIF)

Figure S9 **Dnmt1 Progressively Translocates to the Cytosol in E13.5 PGCs.** (A) Blots of Figure 6A were subjected to densitometry analysis using Quantity One software. (B) Magnification of Dnmt1 staining in E13.5 PGCs. Merge images were obtained by the combination of green signal (Dnmt1) and red signal (Ddx4). Left panels, both of male and female PGCs, show that some cells still retained Dnmt1 in the nucleus, but in the same cell suspension (right panels), the enzyme was also delocalized at the periphery of the cells. Scale bar, 5 µm. (C) Biochemical separation of nuclear and cytoplasmic fractions of purified E13.5 PGCs showed Dnmt1 mainly in the cytosol. Parp1 and α-Tubulin were used as markers for nuclear and cytoplasmic fractions, respectively. M, male. F, female.
(TIF)

Figure S10 **Expression of** ***Dnmt3*** **Family Genes in PGCs.** qRT-PCR analysis of Dnmt3 family indicated that *Dnmt3a* and *3b* were expressed at all PGC stages while *Dnmt3l* was highly up-regulated in male E13.5 PGCs (mean±s.d., n = 4). Statistically significant differences were determined by One-way ANOVA test followed by Tukey post test (***p<0.001). M, male. F, female.
(TIF)

Figure S11 **PARP Inhibition Does Not Affect Expression of Genes Involved in Maintenance of DNA Methylation Patterns.** (A) Expression analyses of *Dnmt1*, *Pcna* and *Parp1* were performed by qRT-PCR on PGCs purified from control (CTRL) and 3AB-treated AGMs cultured for 72 hrs (mean±s.d., n = 3). No significant differences were obtained using paired Student’s t-test. (B) Immunofluorescence analysis of Dnmt1 protein expression in control (CTRL) and 8 mM 3AB-treated PGCs. Scale bar, 10 µm.
(TIF)

Figure S12 **Expression of** ***Tet2*** **and** ***Tet3*** **Genes in PGCs and after PARP inhibition.** (A) qRT-PCR analysis of *Tet2* and *Tet3* genes carried out on purified PGCs at different developmental stages (mean±s.d., n = 3). Statistically significant differences were determined by One-way ANOVA test followed by Tukey post test (*p<0.05; **p<0.01). (B) Expression analysis performed by qRT-PCR on PGCs purified from AGMs cultured for 72 hrs with/without 3AB. (mean±s.d., n = 3). Statistically significant differences were determined by paired Student’s t-test (*p<0.05). M, male. F, female.
(TIF)

Table S1 **Primers for qRT-PCR Analysis.** TaqMan ID and primer sequences used for SYBR gene expression assays are listed.
(DOC)

Table S2 **Primers for DNA Methylation Analysis.** Primer sequences used for bisulfite sequencing DNA mathylation analysis are listed.
(DOC)
