# Supplementary figures and images for: Poly(ADP-ribosyl)ation Acts in the DNA Demethylation of Mouse Primordial Germ Cells Also with DNA Damage-Independent Roles
Source: PLoS One. 2012 Oct 5;7(10):e46927. doi: 10.1371/journal.pone.0046927 (PMC3465317; doi:10.1371/journal.pone.0046927)

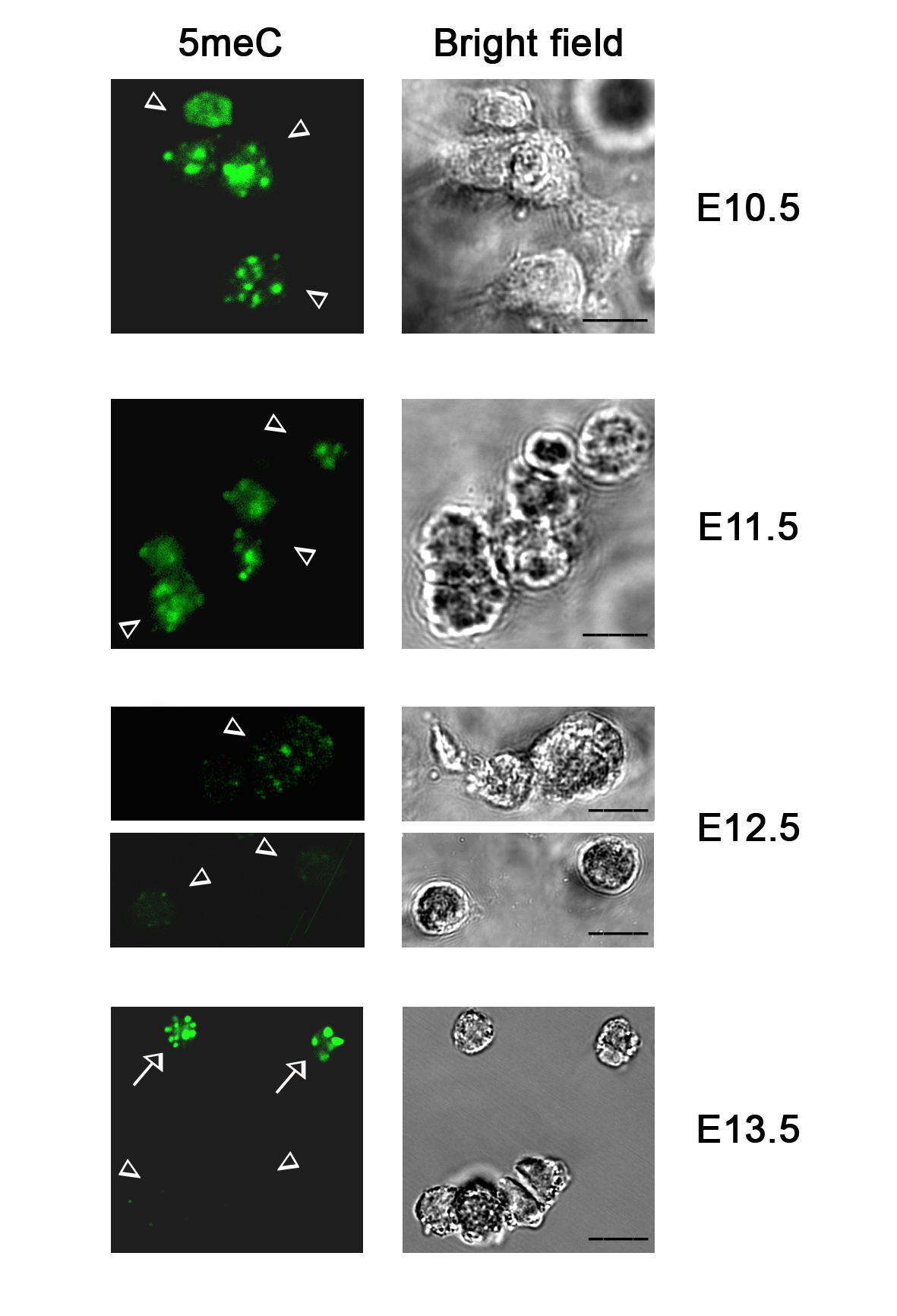

Supplement: Figure S1 — DNA Demethylation Dynamics of PGCs Deriving From CD-1 Mouse Embryos. Immunofluorescence analysis performed with anti-5meC in E10.5–E13.5 PGCs showing gradual loss of DNA methylation. Arrowheads indicate PGCs while arrows indicate SCs. Scale bar, 10 µm. (TIF) [file pone.0046927.s001.tif]

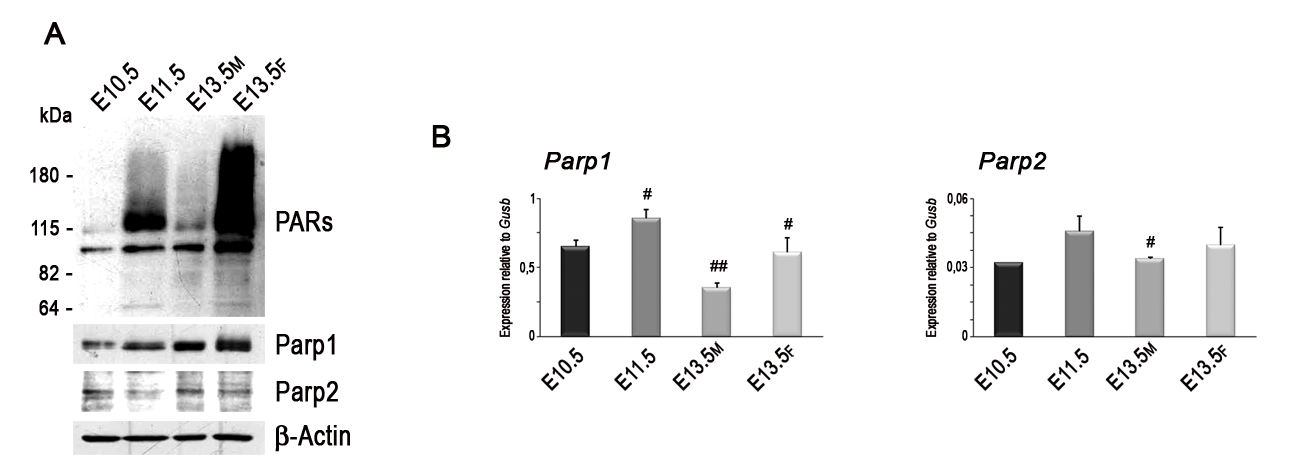

Supplement: Figure S2 — Gonadal Somatic Cells Exhibit Different PARylation Profile in Comparison to PGCs. (A) Western blot analysis performed on control SCs. (B) qRT-PCR analysis carried out on SCs (mean±s.d., n = 3). Unpaired Student’s t-test was performed to compare expression of Parp1 and Parp2 between SCs and PGCs in Figure 1B at each developmental stage (#p<0.05; ##p<0.01). M, male. F, female. (TIF) [file pone.0046927.s002.tif]

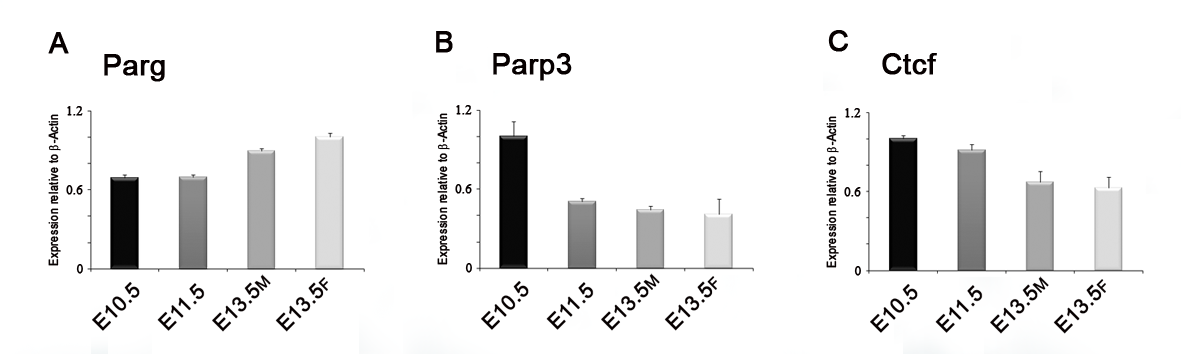

Supplement: Figure S3 — Densitometric Analysis of Parg, Parp3 and Ctcf Protein Expression in PGCs. (A-C) Blots of Figure 2D were subjected to densitometry analysis using Quantity One software. (TIF) [file pone.0046927.s003.tif]

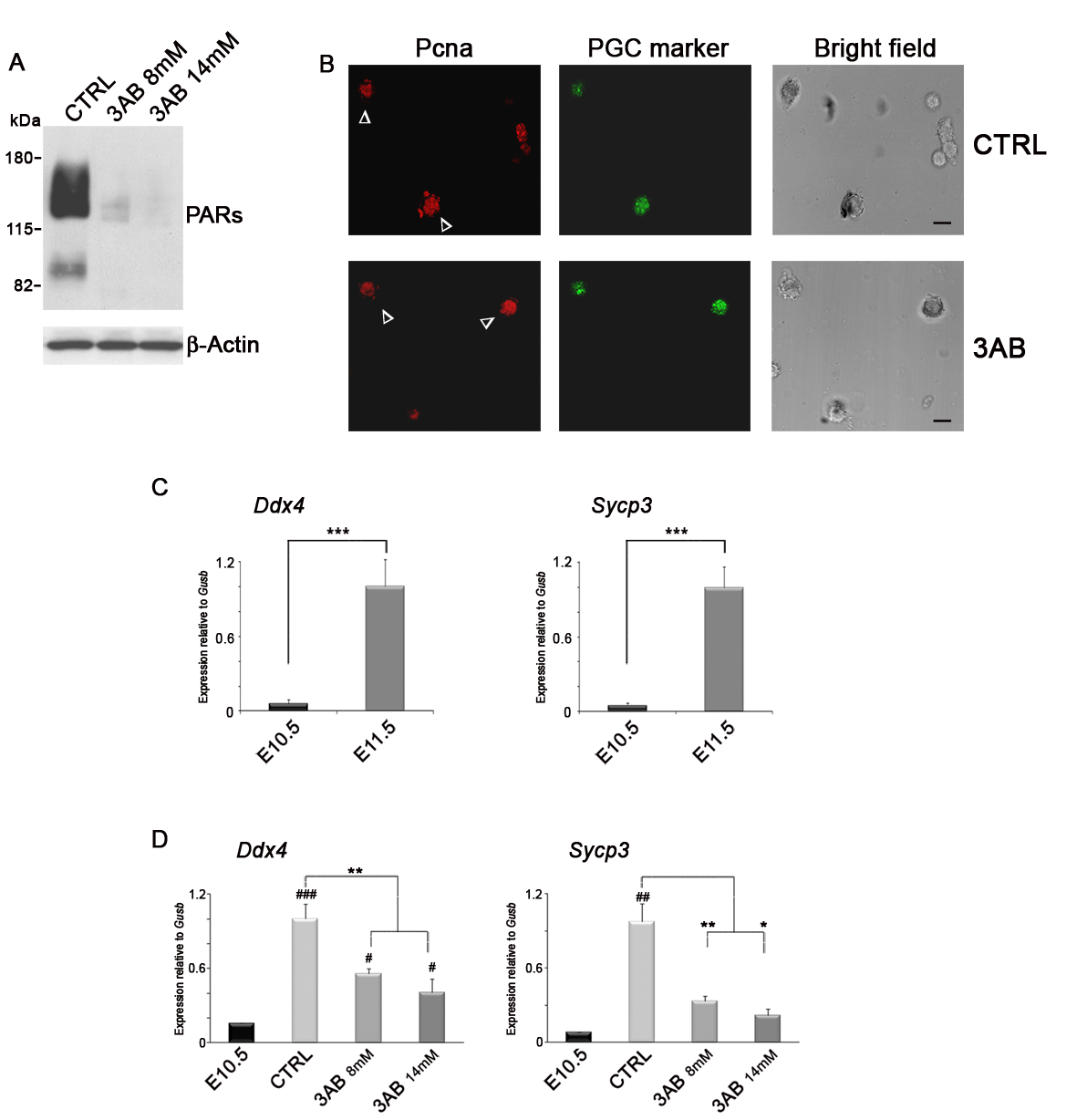

Supplement: Figure S4 — PARP Inhibition Affects the Expression of Germ Cell-specific Genes. (A) Western blot analysis showing ablation of PAR levels in E10.5 AGMs cultured for 72 hrs with 3AB. (B) Immunofluorescence analysis of Pcna expression in control (CTRL) and 8 mM 3AB-treated PGCs. Arrowheads indicate PGCs. Scale bar, 10 µm. (C) qRT-PCR of Ddx4 and Sycp3 genes performed on PGCs prior to (E10.5) and at the beginning (E11.5) of DNA demethylation (mean±s.d., n = 3). Statistically significant differences were determined by unpaired Student’s t-test (***p<0.001). (D) Expression analysis of Ddx4 and Sycp3 performed by qRT-PCR on PGCs purified from control (CTRL) or 3AB-treated AGMs cultured for 72 hrs. E10.5 identifies PGCs purified from not cultured E10.5 AGMs. (mean±s.d., n = 3). One-way ANOVA test followed by Tukey post test was used to determine statistical differences between CTRL and 3AB-treated PGCs (*p<0.05; **p<0.01) as well as between treated/untreated PGCs with E10.5 PGCs (#p<0.05; ##p<0.01; ###p<0.001). (TIF) [file pone.0046927.s004.tif]

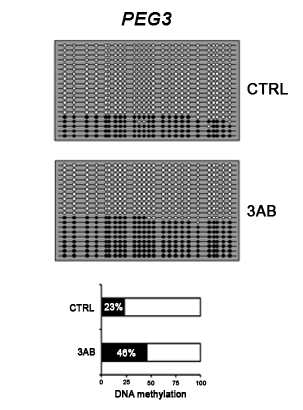

Supplement: Figure S5 — PARP Inhibition Affects DNA Demethylation of the Imprinted Locus Peg3. Bisulfite sequencing analysis of Peg3 DNA methylation performed on PGCs purified from cultured E10.5 AGMs for 72 hrs (CTRL = Control and 3AB). 8 mM 3AB was used for treatment. Each line represents a unique DNA clone; filled and open circles represent methylated and unmethylated CpGs, respectively. Histograms represent the percentage of methylated CpGs. (TIF) [file pone.0046927.s005.tif]

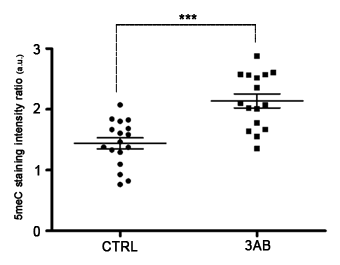

Supplement: Figure S6 — PARP Inhibition Impairs Global DNA Demethylation. Quantification of 5meC fluorescence reported in Figure 4B is here shown as a ratio between 5meC signal recovered from PGCs relative to the average signal from SCs (mean±s.e.m.). Statistically significant differences were determined by Mann-Whitney test (***p<0.001). a.u. = arbitrary unit. (TIF) [file pone.0046927.s006.tif]

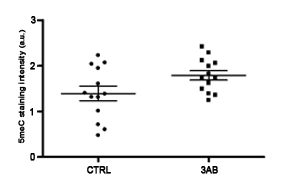

Supplement: Figure S7 — DNA Methylation Levels in PGCs After 48 hrs of 3AB Treatment. Quantification of 5meC staining performed on CRTL and 8 mM 3AB-treated PGCs cultured for 48 hrs (mean±s.e.m.) showing the same trend as observed after 72 hrs of culture. (TIF) [file pone.0046927.s007.tif]

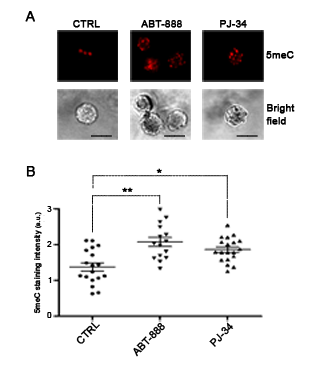

Supplement: Figure S8 — Specific Inhibitors of PARylation Confirm Data Obtained With 3AB. (A) Representative images of control (CTRL), ABT-888 and PJ-34-treated PGCs deriving from AGMs cultured for 72 hrs. Scale bar, 10 µm. (B) Quantification of 5meC staining evidencing that additional inhibitors of PARP activity also maintained high levels of global 5meC (mean±s.e.m.). Statistically significant differences were determined by Kruskal-Wallis test followed by Dunns post test (*p<0.05; **p<0.01). a.u. = arbitrary unit. (TIF) [file pone.0046927.s008.tif]

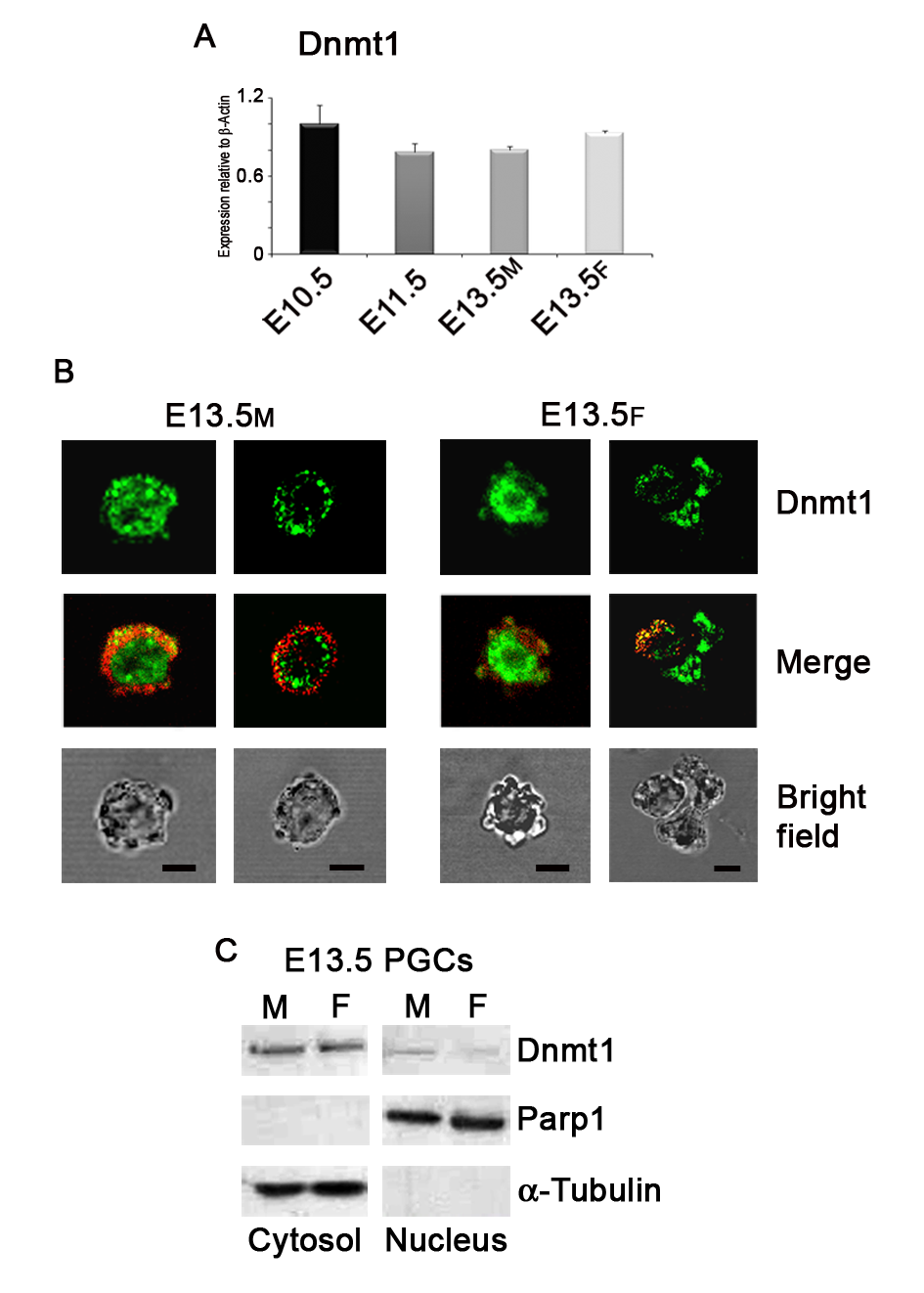

Supplement: Figure S9 — Dnmt1 Progressively Translocates to the Cytosol in E13.5 PGCs. (A) Blots of Figure 6A were subjected to densitometry analysis using Quantity One software. (B) Magnification of Dnmt1 staining in E13.5 PGCs. Merge images were obtained by the combination of green signal (Dnmt1) and red signal (Ddx4). Left panels, both of male and female PGCs, show that some cells still retained Dnmt1 in the nucleus, but in the same cell suspension (right panels), the enzyme was also delocalized at the periphery of the cells. Scale bar, 5 µm. (C) Biochemical separation of nuclear and cytoplasmic fractions of purified E13.5 PGCs showed Dnmt1 mainly in the cytosol. Parp1 and α-Tubulin were used as markers for nuclear and cytoplasmic fractions, respectively. M, male. F, female. (TIF) [file pone.0046927.s009.tif]

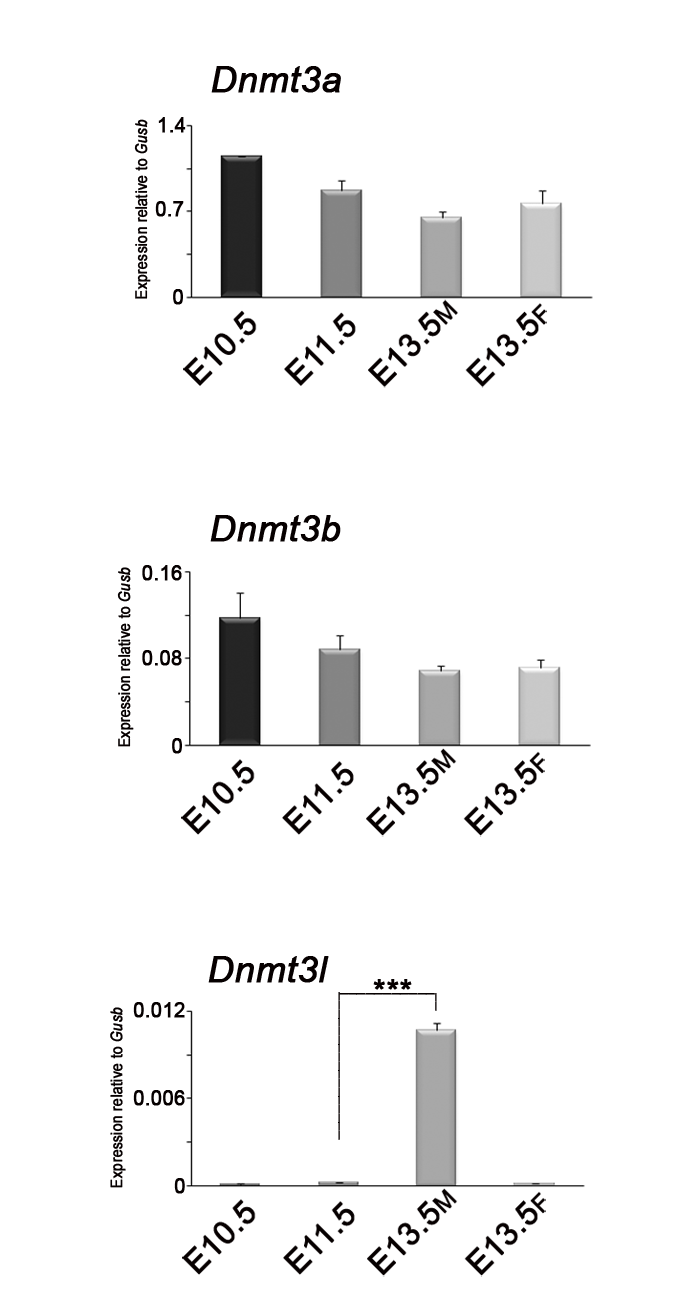

Supplement: Figure S10 — Expression of Dnmt3 Family Genes in PGCs. qRT-PCR analysis of Dnmt3 family indicated that Dnmt3a and 3b were expressed at all PGC stages while Dnmt3l was highly up-regulated in male E13.5 PGCs (mean±s.d., n = 4). Statistically significant differences were determined by One-way ANOVA test followed by Tukey post test (***p<0.001). M, male. F, female. (TIF) [file pone.0046927.s010.tif]

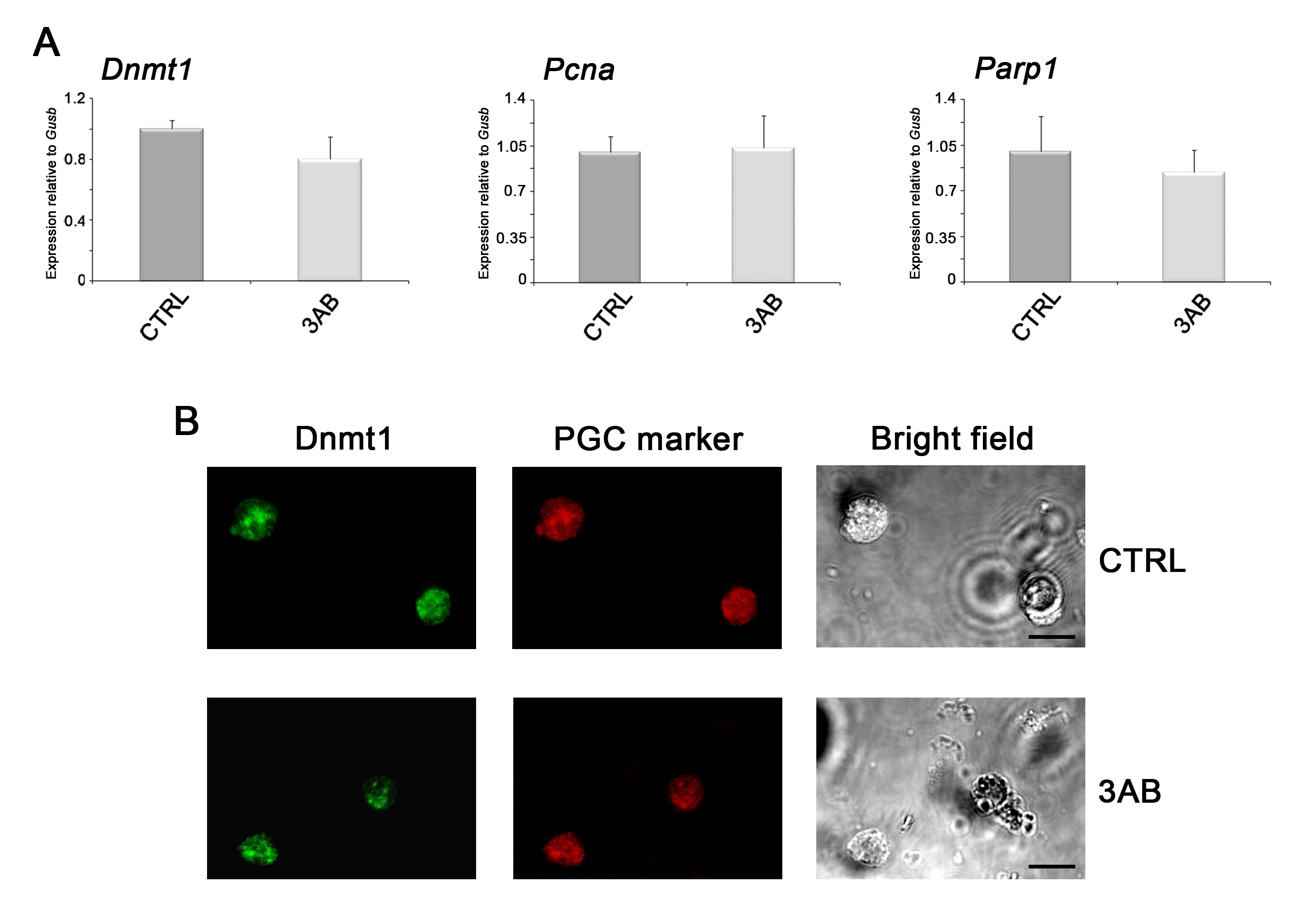

Supplement: Figure S11 — PARP Inhibition Does Not Affect Expression of Genes Involved in Maintenance of DNA Methylation Patterns. (A) Expression analyses of Dnmt1, Pcna and Parp1 were performed by qRT-PCR on PGCs purified from control (CTRL) and 3AB-treated AGMs cultured for 72 hrs (mean±s.d., n = 3). No significant differences were obtained using paired Student’s t-test. (B) Immunofluorescence analysis of Dnmt1 protein expression in control (CTRL) and 8 mM 3AB-treated PGCs. Scale bar, 10 µm. (TIF) [file pone.0046927.s011.tif]

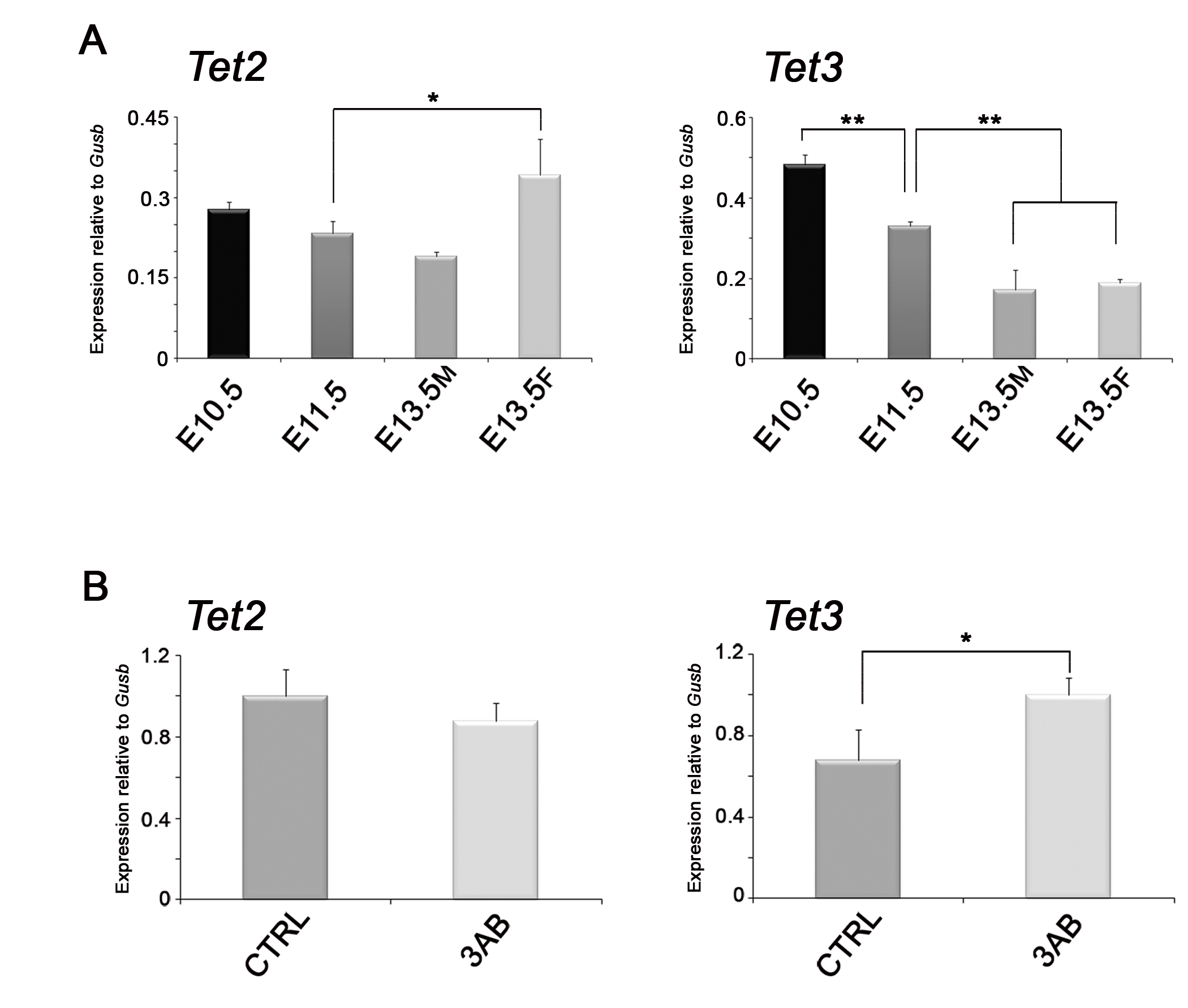

Supplement: Figure S12 — Expression of Tet2 and Tet3 Genes in PGCs and after PARP inhibition. (A) qRT-PCR analysis of Tet2 and Tet3 genes carried out on purified PGCs at different developmental stages (mean±s.d., n = 3). Statistically significant differences were determined by One-way ANOVA test followed by Tukey post test (*p<0.05; **p<0.01). (B) Expression analysis performed by qRT-PCR on PGCs purified from AGMs cultured for 72 hrs with/without 3AB. (mean±s.d., n = 3). Statistically significant differences were determined by paired Student’s t-test (*p<0.05). M, male. F, female. (TIF) [file pone.0046927.s012.tif]
