# Supplementary material for: Poly(ADP-ribosyl)ation Acts in the DNA Demethylation of Mouse Primordial Germ Cells Also with DNA Damage-Independent Roles
Source: PLoS One. 2012 Oct 5;7(10):e46927. doi: 10.1371/journal.pone.0046927 (PMC3465317; doi:10.1371/journal.pone.0046927)
Supplement: Table S1 — Primers for qRT-PCR Analysis. TaqMan ID and primer sequences used for SYBR gene expression assays are listed. (DOC) [file pone.0046927.s013.doc]

**Table S1. Primers for qRT-PCR Analysis.**

| TaqMan Gene Expression Assays |
| --- |
| | Gene | [Assay ID](https://products.appliedbiosystems.com:443/ab/en/US/adirect/ab?cmd=ABGEKeywordResults&adv_phrase3=EXACT&adv_phrase2=EXACT&adv_phrase1=EXACT&assayType=GE&catID=601267&SearchRequest.Common.SortSpec=ACC+asc&adv_kw_filter3=ALL&adv_kw_filter2=ALL&SearchRequest.Common.QueryText=gusb&kwdropdown=ge&adv_kw_filter1=ALL&species=Mus+musculus&adv_query_text3=&searchType=keyword&adv_query_text2=&adv_query_text1=&uploadType=ID+List&adv_boolean3=AND&adv_boolean2=AND&adv_boolean1=AND&chkBatchQueryText=false&kwfilter=ALL&) | | --- | --- | | *Parp1* | Mm00500154_m1 | | *Parp2* | Mm00456462_m1 | | *Parp3* | Mm00467486_m1 | | *Parg* | Mm00449466_m1 | | *Ctcf* | Mm00484027_m1 | | *Dnmt1* | Mm00599763_m1 | | *Dnmt3a* | Mm00432871_g1 | | *Dnmt3b* | Mm01240113_m1 | | *Dnmt3l* | Mm00457635_m1 | | *Ddx4* | Mm00802445_m1 | | *Sycp3* | Mm00488519_m1 | | *Gusb* | [Mm00446956_m1](https://products.appliedbiosystems.com:443/ab/en/US/adirect/ab?cmd=ABAssayDetailDisplay&assayID=Mm00446956_m1&Fs=y&adv_phrase3=EXACT&adv_phrase2=EXACT&adv_phrase1=EXACT&assayType=GE&catID=601267&adv_kw_filter3=ALL&srchType=keyword&adv_kw_filter2=ALL&SearchRequest.Common.QueryText=gusb&kwdropdown=ge&adv_kw_filter1=ALL&species=Mus+musculus&adv_query_text3=&searchType=keyword&adv_query_text2=&adv_query_text1=&uploadType=ID+List&adv_boolean3=AND&adv_boolean2=AND&adv_boolean1=AND&chkBatchQueryText=false&kwfilter=ALL&SearchRequest.Common.PageNumber=1&msgType=ABGEKeywordResults) | | *Gapdh* | Mm99999915_g1 | | *B2m* | Mm00437764_m1 | | *Hprt1* | Mm00446968_m1 | |

| SYBR Green Gene Expression Primers |
| --- |
| | Gene | Sense | Antisense | | --- | --- | --- | | *Uhrf1* | CCCAGGTGGTCCAGGTACAG | CACGAGCACGGACATTCTTG | | *Pcna* | gggctgaagataatgcagaca | GGATTCCAAGTTGCTCCACA | | *Dmap1* | Tgcaggcatcaagtttccag | cagctcctctgtgggggtag | | *Tet1* | tttggttcgtgagcgtgtag | tgcaggtacgctttttgttg | | *Tet2* | aacctggctactgtcattgctcca | agatgttctgctggtctctgtgggaa | | *Tet3* | ccggattgagaaggtcatctac | aagataacaatcacgggcgttct | | *Aicda* | GATAGTGCCACCTCCTGCTC | GCGTAGGAACAACAATTCCAC | | *Gusb* | ACTGACACCTCCATGTATCCCAAG | CAGTAGGTCACCAGCCCGATG | |
