# Supplementary material for: Poly(ADP-ribosyl)ation Acts in the DNA Demethylation of Mouse Primordial Germ Cells Also with DNA Damage-Independent Roles
Source: PLoS One. 2012 Oct 5;7(10):e46927. doi: 10.1371/journal.pone.0046927 (PMC3465317; doi:10.1371/journal.pone.0046927)
Supplement: Table S2 — Primers for DNA Methylation Analysis. Primer sequences used for bisulfite sequencing DNA mathylation analysis are listed. (DOC) [file pone.0046927.s014.doc]

**Table S2. Primers for DNA Methylation Analysis.**

| Bisulfite Sequencing Primers |
| --- |
| | Gene | Sense | Antisense | | --- | --- | --- | | *Ddx4* | TGAATGAATATAATGGAATTGATGAGTT | AAAACAACAAATAACATCAAA | | *Sycp3* | GAATGAGGATTTATGAGTAAAGATGGTT | CCCCCATCTCCTTAACCTCAA | | *H19* | AGGGATTTATAGGGGTGGTAA | AAATACACAAATACCTAATCC | | *Peg3* | tttttagattttgtttgggggtttttaata | aatccctatcacctaaataacatccctaca | |
